# Supplementary material for: A coopetition-driven strategy of parallel/perpendicular aromatic stacking enabling metastable supramolecular polymerization
Source: Nat Commun. 2024 Dec 30;15:10762. doi: 10.1038/s41467-024-55106-z (PMC11686131; doi:10.1038/s41467-024-55106-z)
Supplement: Supplementary file 1 — Supplementary Information [file 41467_2024_55106_MOESM1_ESM.pdf]

Supplementary Information

A coopetition-driven strategy of parallel/perpendicular aromatic stacking enabling metastable supramolecular polymerization

Zhao Gao,[1,§] Xuxu Xie,[1,§] Juan Zhang,[1] Wei Yuan,[2] Hongxia Yan[1], and Wei Tian*,[1]

[1]School of Chemistry and Chemical Engineering, Northwestern Polytechnical University, Xi'an, 710072, China
[2]Division of Chemistry and Biological Chemistry, School of Physical and Mathematical Sciences, Nanyang Technological University, Singapore 637371, Singapore
[§]The two authors contributed equally to this work.
*Correspondence and requests for materials should be addressed to W.T. (email: happytw_3000@nwpu.edu.cn).

1. Crystallographic data of 1–4

Supplementary Table 1. Crystal data and structure refinement for **1AggII**.

Identification code	**1AggII**
CCDC number	2351020
ORTEP structure	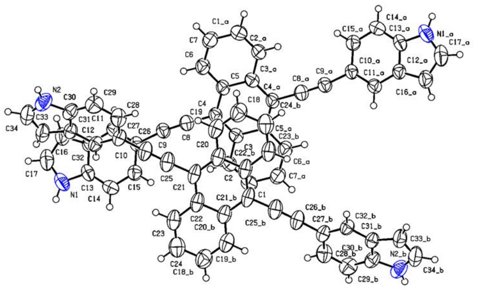
Empirical formula	$C_{34}H_{20}N_2$
Formula weight	456.52
Temperature [K]	193
Crystal system	monoclinic
Space group	$P2_1/n$
a [Å]	9.4009(6)
b [Å]	27.532(2)
c [Å]	9.5943(7)
α [°]	90
β [°]	109.583(2)
γ [°]	90
Volume [Å^3]	2339.6(3)
Z	4
ρ_{calc} [g cm^{-3}]	1.296
μ [mm^{-1}]	0.076
F(000)	952
Crystal size [mm^3]	0.12 × 0.1 × 0.09
Radiation	MoKα (λ = 0.71073)
2θ range for data collection [°]	4.742 to 52.742
Index ranges	$-11 \le h \le 11, -34 \le k \le 27, -11 \le l \le 11$
Reflections collected	18842
Independent reflections	4775 [R_{int} = 0.0743, R_{sigma} = 0.0708]
Data/restraints/parameters	4775/0/325
Goodness-of-fit on F^2	1.03
Final R indexes [$I \geqslant 2\sigma(I)$]	R_1 = 0.0810, wR_2 = 0.1642
Final R indexes [all data]	R_1 = 0.1566, wR_2 = 0.2018
Largest diff. peak/hole [e Å^{-3}]	0.43/-0.19

Supplementary Table 2. Crystal data and structure refinement for **2AggII**.

Identification code	**2AggII**
CCDC number	2351018
ORTEP structure	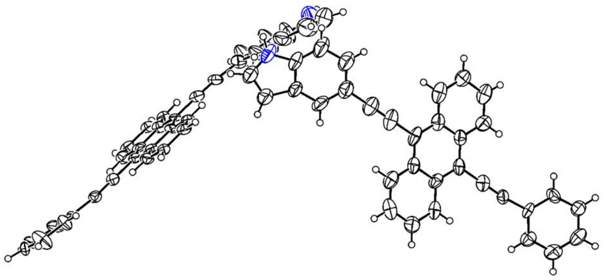
Empirical formula	$C_{32}H_{19}N$
Formula weight	417.48
Temperature [K]	100
Crystal system	monoclinic
Space group	Cc
a [Å]	50.413(14)
b [Å]	5.1748(13)
c [Å]	16.624(4)
α [°]	90
β [°]	90.349(17)
γ [°]	90
Volume [Å^3]	4336.6(19)
Z	8
ρ_{calc} [g cm^{-3}]	1.279
μ [mm^{-1}]	0.074
F(000)	1744
Crystal size [mm^3]	0.16 × 0.12 × 0.1
Radiation	MoKα (λ = 0.71073)
2θ range for data collection [°]	4.848 to 50.054
Index ranges	-60 ⩽ h ⩽ 59, -6 ⩽ k ⩽ 6, -19 ⩽ l ⩽ 18
Reflections collected	15892
Independent reflections	7300 [R_{int} = 0.1377, R_{sigma} = 0.2582]
Data/restraints/parameters	7300/147/571
Goodness-of-fit on F^2	1.027
Final R indexes [$I \geqslant 2\sigma(I)$]	R_1 = 0.0952, wR_2 = 0.2016
Final R indexes [all data]	R_1 = 0.2412, wR_2 = 0.2560
Largest diff. peak/hole [e Å^{-3}]	0.35/-0.60
Note on a B-level alert	The B-level alert is the low bond precision on C-C bonds. Response: the quality of the tested crystal is poor, and the resolution of the obtained crystal data is not high. Restrictions have been imposed on the C-C bond during the refinement process.

Supplementary Table 3. Crystal data and structure refinement for **3AggII**.

Identification code	**3AggII**
CCDC number	2368744
ORTEP structure	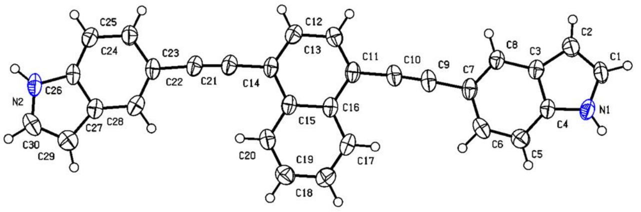
Empirical formula	$C_{30}H_{18}N_2$
Formula weight	406.46
Temperature [K]	193.15
Crystal system	monoclinic
Space group	$P2_1/c$
a [Å]	7.8497(7)
b [Å]	18.3432(12)
c [Å]	14.5056(12)
α [°]	90
β [°]	99.243(3)
γ [°]	90
Volume [Å^3]	2061.5(3)
Z	4
ρ_{calc} [g cm^{-3}]	1.31
μ [mm^{-1}]	0.077
F(000)	848
Crystal size [mm^3]	0.74 × 0.63 × 0.42
Radiation	MoKα (λ = 0.71073)
2*θ* range for data collection [°]	4.442 to 50.05
Index ranges	$-9 \leqslant h \leqslant 9, -21 \leqslant k \leqslant 21, -17 \leqslant l \leqslant 17$
Reflections collected	40279
Independent reflections	3646 [R_{int} = 0.1105, R_{sigma} = 0.0548]
Data/restraints/parameters	3646/0/289
Goodness-of-fit on F^2	1.052
Final *R* indexes [$I \geqslant 2\sigma(I)$]	R_1 = 0.0648, wR_2 = 0.1820
Final *R* indexes [all data]	R_1 = 0.0828, wR_2 = 0.1983
Largest diff. peak/hole [e Å^{-3}]	0.54/-0.23

Supplementary Table 4. Crystal data and structure refinement for **4**.

Identification code	**4**
CCDC number	2368743
ORTEP structure	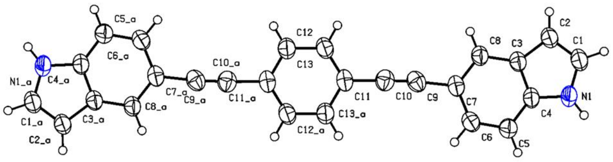
Empirical formula	$C_{26}H_{16}N_2$
Formula weight	356.41
Temperature [K]	193
Crystal system	monoclinic
Space group	$P2_1/c$
a [Å]	7.6761(5)
b [Å]	16.4290(10)
c [Å]	7.6609(5)
α [°]	90
β [°]	112.694(4)
γ [°]	90
Volume [Å^3]	891.32(10)
Z	2
ρ_{calc} [g cm^{-3}]	1.328
μ [mm^{-1}]	0.604
F(000)	372
Crystal size [mm^3]	0.12 × 0.11 × 0.08
Radiation	CuKα (λ = 1.54178)
2θ range for data collection [°]	10.77 to 149.606
Index ranges	$-9 \leqslant h \leqslant 9, -20 \leqslant k \leqslant 16, -7 \leqslant l \leqslant 9$
Reflections collected	7008
Independent reflections	1793 [R_{int} = 0.0837, R_{sigma} = 0.0599]
Data/restraints/parameters	1793/0/128
Goodness-of-fit on F^2	1.122
Final *R* indexes [$I \geqslant 2\sigma(I)$]	R_1 = 0.0582, wR_2 = 0.1811
Final *R* indexes [all data]	R_1 = 0.0855, wR_2 = 0.2079
Largest diff. peak/hole [e Å^{-3}]	0.39/-0.18

2. Supplementary Figures and Notes

We have selected most of the common solvents to study the effect of solvents on the self-assembly of **1**. As shown in Supplementary Figure 1, nearly identical absorption spectra of **1** in DCM, THF, MeCN, and DMF were observed, which proved that monomer **1** possessed relatively good solubility in these solvents (named as **1M**). Also, the absence of Tyndall effect verified no formation of large aggregates. However, when the solvent was changed to H_2O and the mixed solvent DMF/H_2O (1 : 24, *v*/*v*), the absorption spectra were significantly varied, while a Tyndall effect occurred. These results undoubtedly indicated the transition from the monomeric state **1M** to the supramolecular aggregated state of **1** (named as **1AggI**).

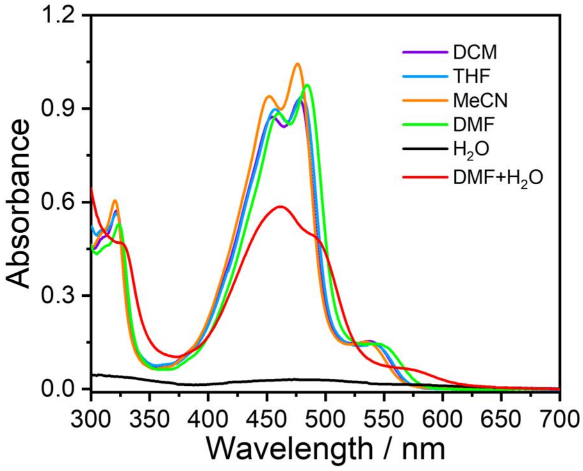

Supplementary Figure 1. Solvent-dependent UV–Vis absorption spectra of **1** ($c = 3.00\times10^{-5}$ M) at 298 K in DCM, THF, MeCN, DMF, H_2O and DMF/H_2O (1 : 24, *v*/*v*).

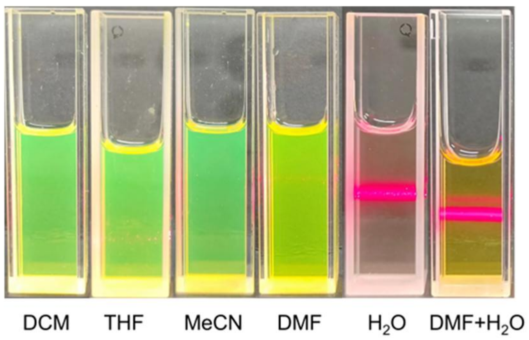

Supplementary Figure 2. Photographs of the Tyndall phenomenon of **1** ($c = 3.00\times10^{-5}$ M) and the corresponding luminescence under UV light at 298 K in DCM, THF, MeCN, DMF, H_2O and DMF/H_2O (1 : 24, *v*/*v*).

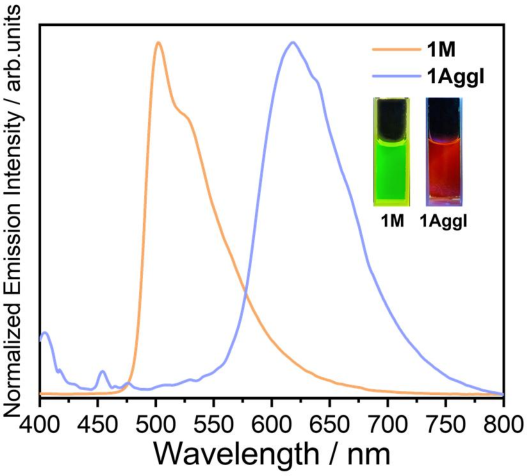

Supplementary Figure 3. Normalized fluorescence spectra of **1** in DMF (**1M**) and the freshly prepared DMF/H_2O (1 : 24, *v*/*v*) solution of **1** (**1AggI**). λ_{ex} = 365 nm. Inset: photographs of **1M** and **1AggI** under UV light. λ_{ex} = 365 nm

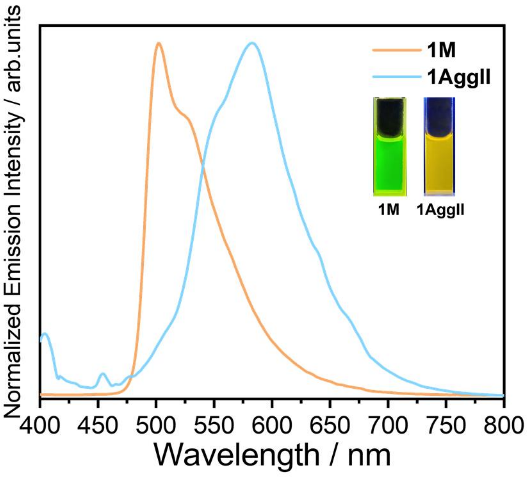

Supplementary Figure 4. Normalized fluorescence spectra of **1M** and **1AggII**. λ_{ex} = 365 nm. **1AggII** was obtained by slowly cooling the DMF/H_2O (1 : 24, *v*/*v*) solution of **1** at a rate of 0.5 K min^{-1} to 298 K. Inset: photographs of **1M** and **1AggII** under UV light. λ_{ex} = 365 nm.

The supramolecular polymerization mechanism can be probed *via* temperature-dependent UV–Vis spectra measurements.[1] For monomers **1**–**2**, non-sigmoidal curves were obtained. The fitted parameters include enthalpy of elongation (ΔH_e), enthalpy of nucleation (ΔH_n), entropy (ΔS), and nucleation penalty (*NP*). $\Delta G = \Delta H_e - T\Delta S$. These parameters are related to the equilibrium constants

of the elongation phase (K_e) and nucleation phase (K_n) by using Eq. (1) and Eq. (2):

$$K_e = exp\left(-\frac{\Delta G}{RT}\right) \qquad \text{Eq. (1)}$$

$$K_n = exp\left(-\frac{NP}{RT}\right) \times K_e \qquad \text{Eq. (2)}$$

In this equation, $NP = \Delta H_e - \Delta H_n$.

The lhsdesign function in Matlab was used to solve the above equations. ΔS was sampled between –0.01 and –1.00 kJ·mol^{-1}·K^{-1}, ΔG was set between –20 and –300 kJ·mol^{-1} to determine the enthalpy and NP between –1 and –35 kJ·mol^{-1}. Levenberg-Marquardt algorithm is used to optimize each initial parameter set to minimize the sum of squares of the cost vector. Matlab function lsqnonlin is used to minimize the cost vector, and the minimum norm of the residual sum of squares is selected.

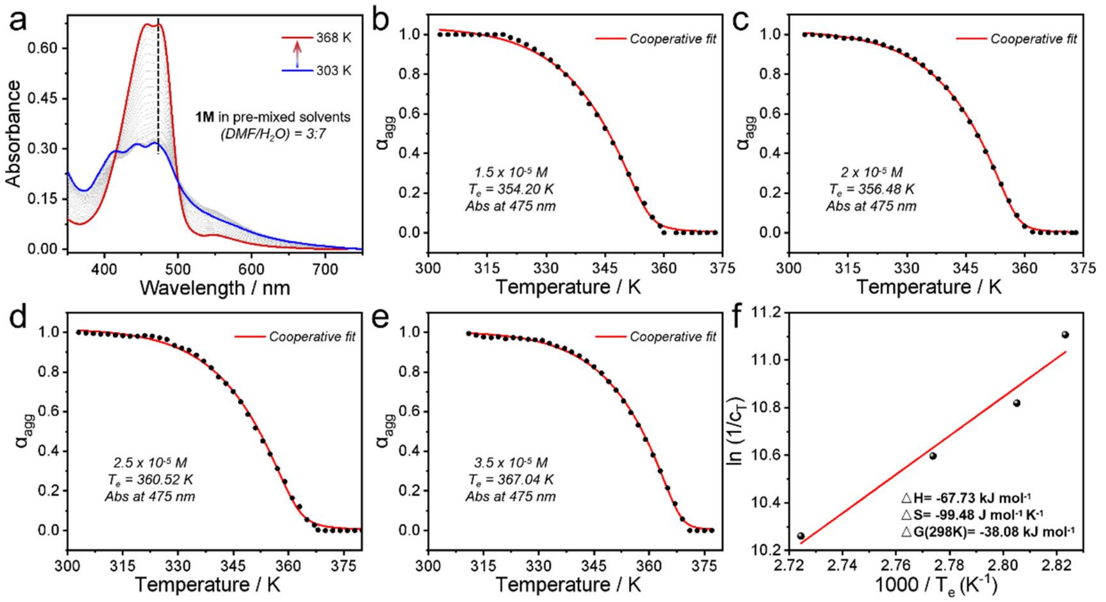

Supplementary Figure 5. (a) Temperature-dependent UV–Vis absorption of **1** in DMF/H_2O (3 : 7, *v*/*v*, c = 3.00×10^{-5} M) upon heating (rate: 2 K/min) from 303 K to 368 K. (b-e) Plot of the variation of the degree of aggregation (α_{agg}) monitored at λ = 475 nm versus temperature at different concentrations. The normalized melting curves at λ = 475 nm display non-sigmoidal curves, which are characteristic for the nucleation-elongation cooperative assembling mechanism. (d) Van't Hoff plot of **1AggII**. The red line denotes the respective linear fitting curve. According to the Van’t Hoff plot, ΔG of the assembly process of **1AggII** was calculated to be -38.08 kJ mol^{-1} at 298 K.

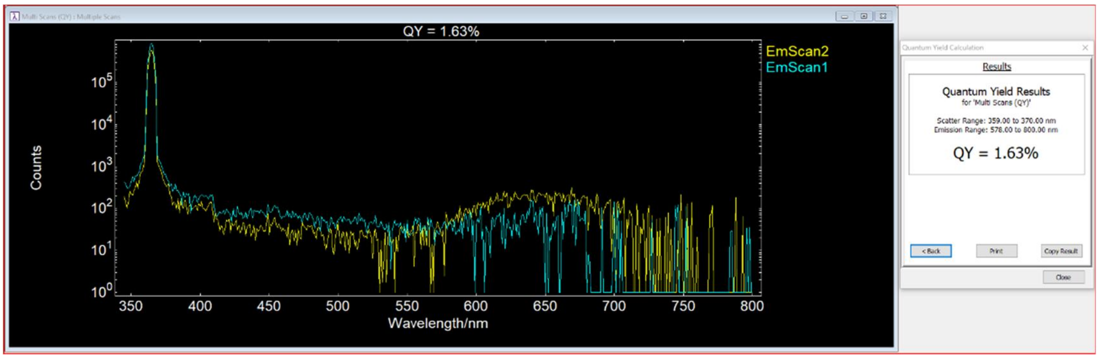

Supplementary Figure 6. Absolute fluorescence quantum yield of **1AggI** in DMF/H_2O (1 : 24, *v*/*v*, *c* = 3.00×10^{-5} M).

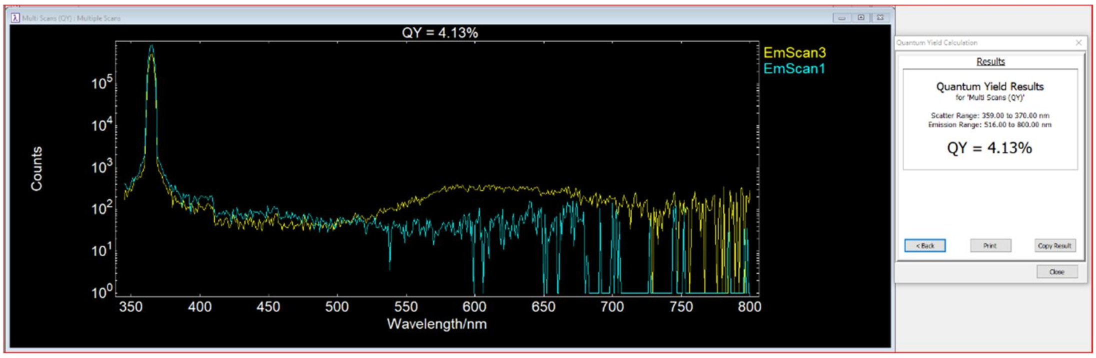

Supplementary Figure 7. Absolute fluorescence quantum yield of **1AggII** in DMF/H_2O (1 : 24, *v*/*v*, *c* = 3.00×10^{-5} M).

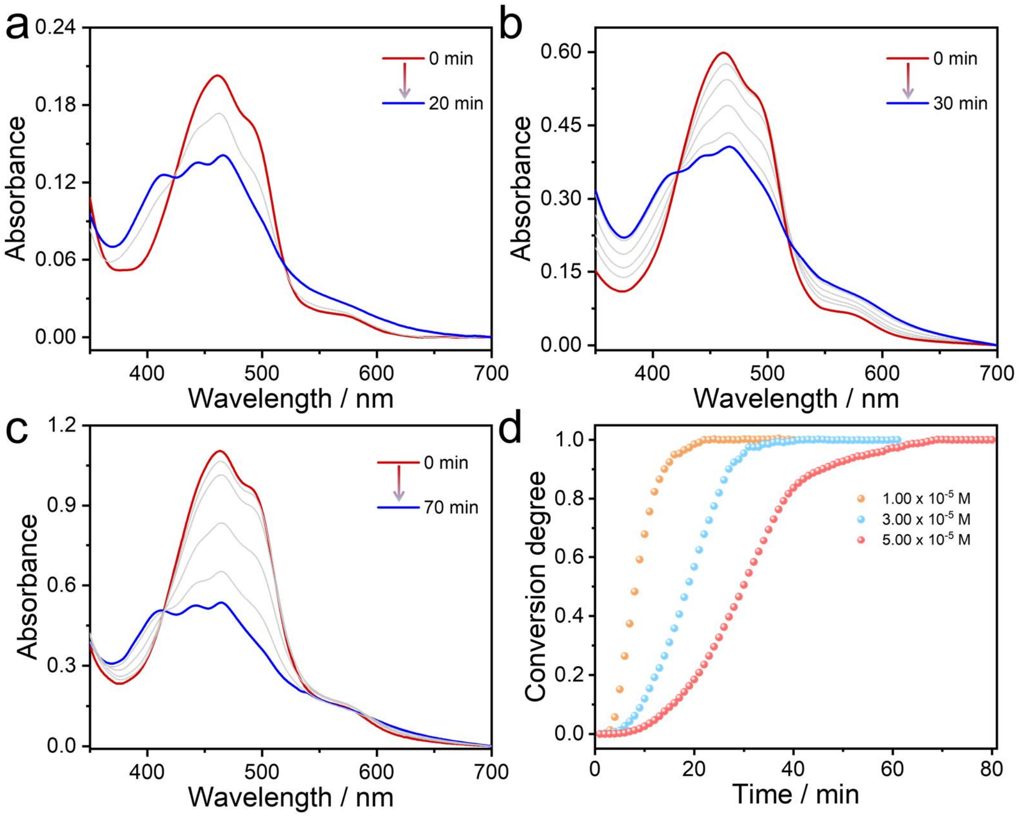

Supplementary Figure 8. Time-dependent UV–Vis measurements following the transformation from **1AggI** into **1AggII** in DMF/H_2O (1:24, *v*/*v*) at 298 K at different concentrations: (a) 1.00×10^{-5} M, (b) 3.00×10^{-5} M and (c) 5.00×10^{-5} M. (d) Plot of the normalized absorption at λ = 463 nm against time. Upon increasing concentration, a slower transformation is observed, indicating that the transformation from **1AggI** into **1AggII** follows an off-pathway mechanism.

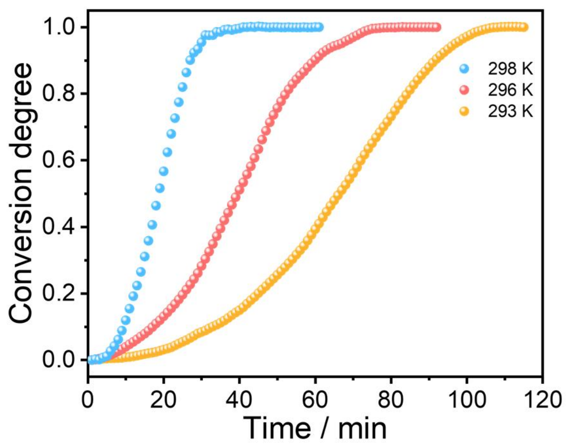

Supplementary Figure 9. Kinetic profiles of the transformation of **1AggI** into **1AggII** (*c* = 3.00×10^{-5} M) at different temperature.

Kinetic fitting of nucleation-elongation process: the conversion degree from **1AggI** to **1AggII** versus time was fitted well with the Finke–Watzky (F–W) model[2] by using Eq. (3), resulting in the nucleation (k_n) and elongation (k_e) rate constants.

$$[c]_t = [c]_0 \times \left(1 - \frac{k_n + k_e[c]_0}{k_e[c]_0 + k_n \exp(k_n t + k_e[c]_0 t)}\right) \quad \text{Eq. (3)}$$

In this equation, $[c]_0$ is the initial concentration.

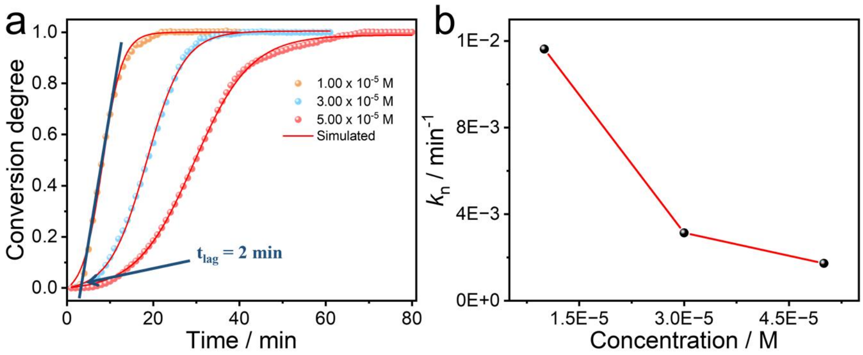

Supplementary Figure 10. (a) Fits of concentration-dependent kinetic data of **1** in DMF/H_2O (1:24, *v*/*v*) at 298 K to Finke–Watzky model. (b) Nucleation rate constant (k_n) of **1** versus concentration. Conversion.

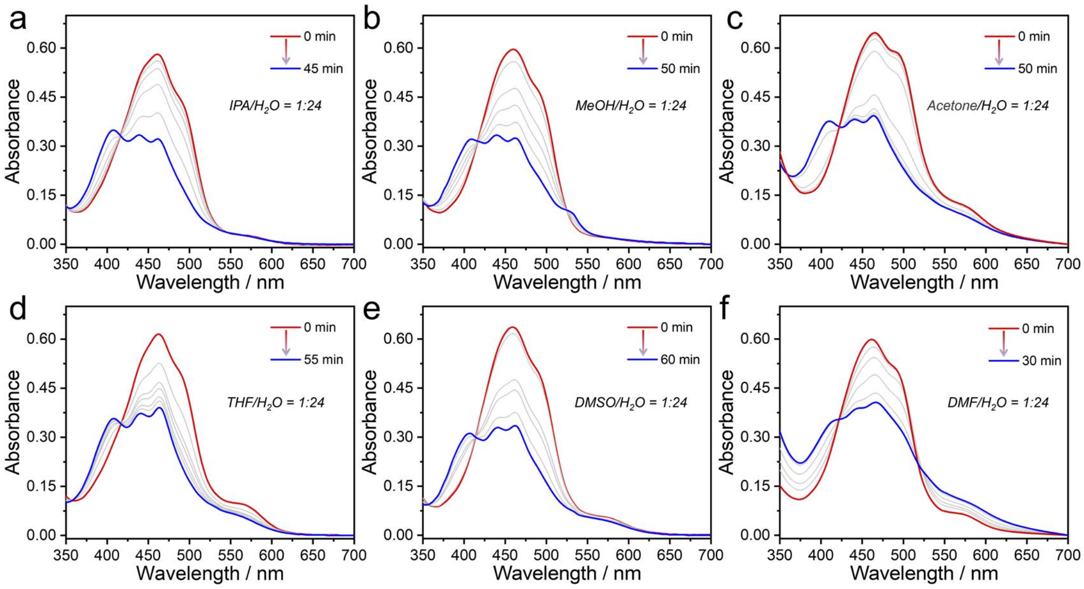

Supplementary Figure 11. Time-dependent UV–Vis spectra measurements following the transformation from **1AggI** ($c = 3.00\times10^{-5}$ M) to **1AggII** in (a) IPA/H_2O (1 : 24, *v*/*v*), (b) MeOH/H_2O (1 : 24, *v*/*v*), (c) Acetone/H_2O (1 : 24, *v*/*v*), (d) THF/H_2O (1 : 24, *v*/*v*), (e) DMSO/H_2O (1 : 24, *v*/*v*) and (f) DMF/H_2O (1 : 24, *v*/*v*) at 298 K.

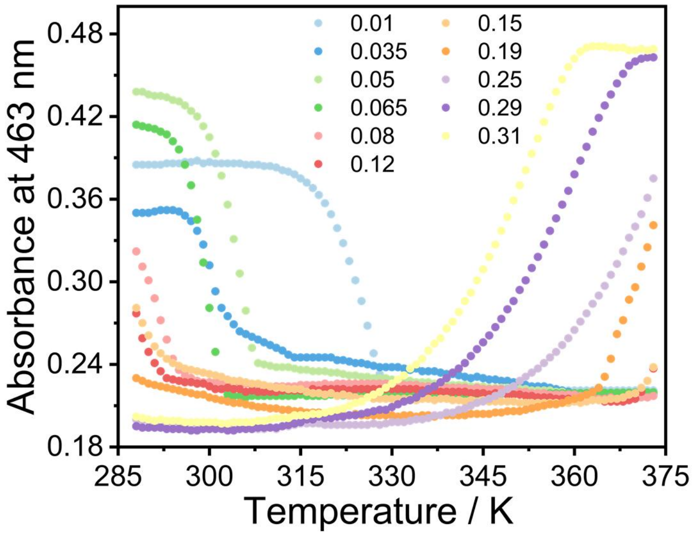

Supplementary Figure 12. Temperature-dependent absorption spectra of **1** (c = 2.00×10^{-5} M) separately monitored at 463 nm in H_2O/DMF mixtures with different DMF volume fraction ranging from 1% to 31%.

Supplementary Table 5. Critical temperature obtained from the temperature-dependent denaturation experiments of **1** in H_2O/DMF mixtures with different DMF volume fraction.

DMF volume fraction	T_{AggI} (K)	$T_{\mathrm{AggI\text{-}AggII}}$ (K)	$T_{\mathrm{AggII\text{-}Mono}}$ (K)	T_{Mono} (K)
0.01	312	330		
0.035	297	313		
0.05	293	308		
0.065	291	303		
0.08		297		
0.12		293	370	
0.15		292	368	
0.19			352	
0.25			333	
0.29			317	371
0.31			310	362

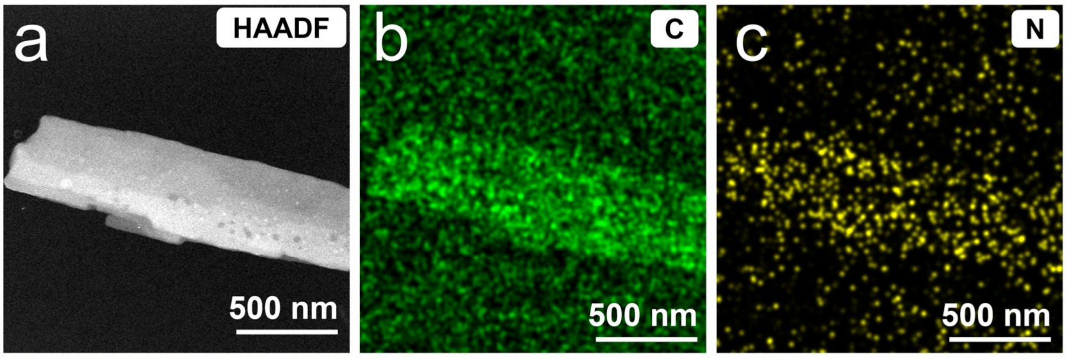

d

Z	Element	Family	Atomic Fraction (%)	Atomic Error (%)	Mass Fraction (%)	Mass Error (%)	Fit error (%)
6	C	K	95.36	6.07	94.63	4.22	3.07
7	N	K	4.64	1.28	5.37	1.46	18.06

Supplementary Figure 13. (a)–(c) HAADF-STEM image and the corresponding EDS for elemental mapping of **1AggII.** (d) The detailed percentage of element distribution from EDS mapping.

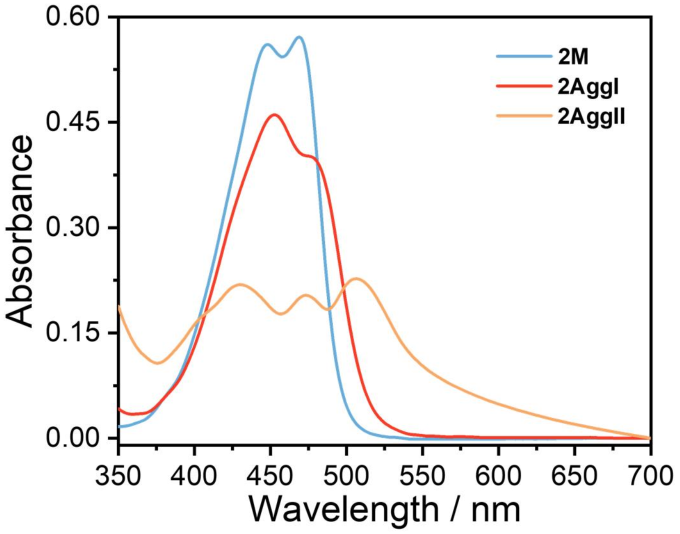

Supplementary Figure 14. UV–Vis absorption spectra of **2** in H_2O/DMF (3:1, *v*/*v*, $c = 3.00\times10^{-5}$ M) at 370 K (blue line, **2M**), fast cooling to 298 K (red line, **2AggI**), and standing **2AggI** at room temperature for 90 min (orange line, **2AggII**).

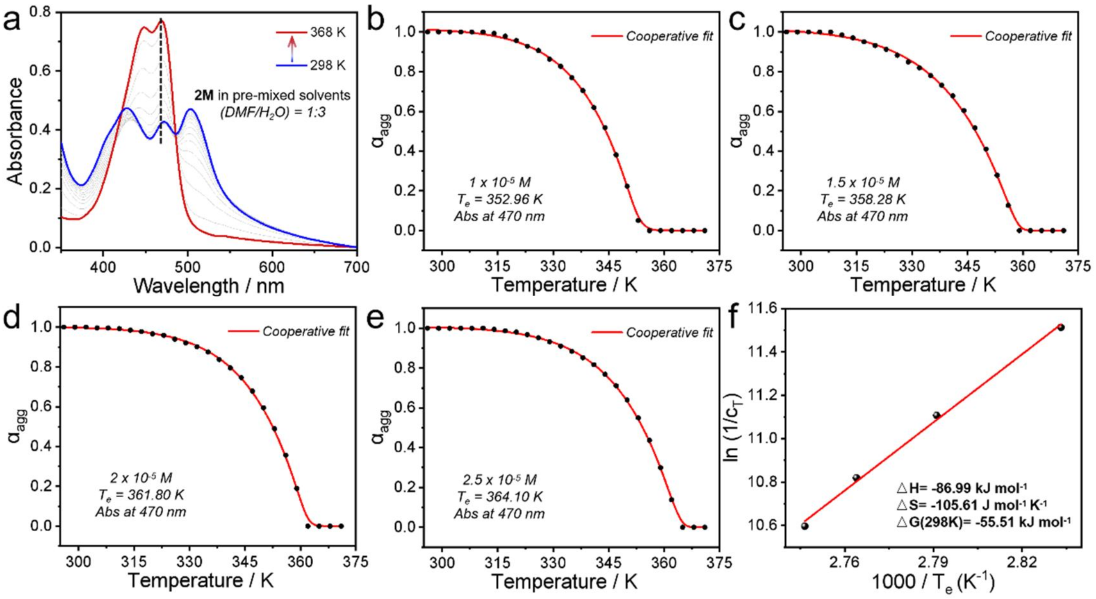

Supplementary Figure 15. (a) Temperature-dependent UV–Vis absorption of **2** in DMF/H_2O (1:3, *v*/*v*, $c = 4.00 \times 10^{-5}$ M) upon cooling (rate: 1 K/min) from 368 K to 298 K. (b-e) Plot of the variation of the degree of aggregation monitored at λ = 470 nm versus temperature at different concentrations. The normalized melting curves at λ = 470 nm display non-sigmoidal curves, which are characteristic for the nucleation-elongation cooperative mechanism. (d) Van't Hoff plot of **2AggII**. The red line denotes the respective linear fitting curve. According to the Van’t Hoff plot, ΔG of the self-assembly process of **2AggII** is calculated to be -55.51 kJ mol^{-1} at 298 K.

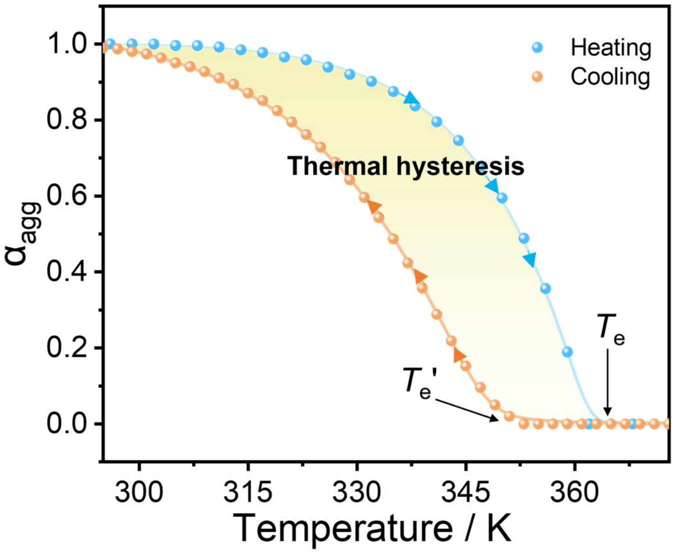

Supplementary Figure 16. Temperature-dependent aggregated degree (α_{Agg}) of **2AggII** calculated from the absorption intensities at λ = 470 nm observed in the heating and cooling processes. The

yellow area represents the thermal hysteresis range for kinetically stabilizing **2M**. The heating curve was not overlapped with the cooling curve, indicating a pronounced hysteresis loop appeared. The curves fitted well with the mathematical model, acquiring the clearly distinguished critical elongation temperature T_e' and T_e as 346.2 K and 361.8 K, respectively.

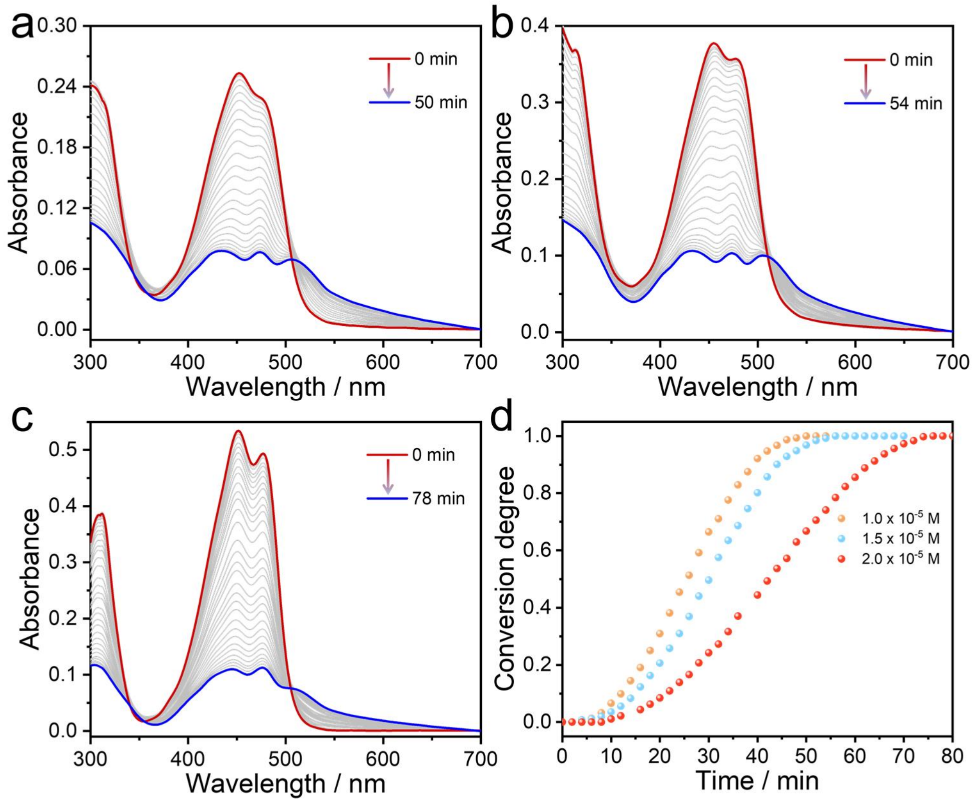

Supplementary Figure 17. Time-dependent UV–Vis measurements following the transformation from **2AggI** into **2AggII** in DMF/H_2O (1:3, *v*/*v*) at 298 K at different concentrations: (a) 1.00×10^{-5} M, (b) 1.50×10^{-5} M and (c) 2.00×10^{-5} M. (d) Plot of the normalized absorption at $\lambda = 450$ nm against time. Upon increasing concentration, a slower transformation is observed, indicating that the transformation from **2AggI** into **2AggII** follows an off-pathway mechanism.

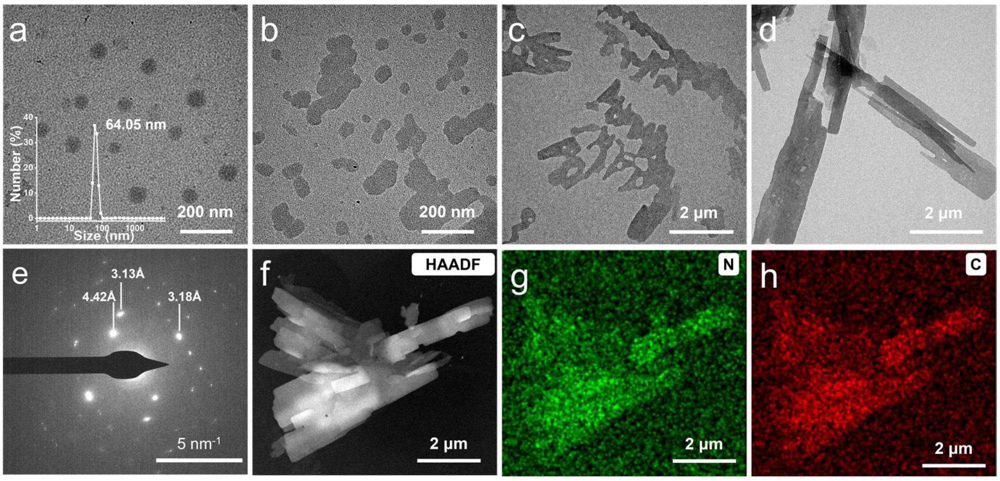

Supplementary Figure 18. Time-dependent TEM images of **2** (5.00×10^{-5} M) in DMF/H_2O (1:3, *v*/*v*) at 298 K for (a) 0 min, (b) 30 min, (c) 60 min and (d) 90 min. Inset of (a): DLS results of **2AggI**. (e) SAED of **2AggII**. (f-h) HAADF-STEM image and corresponding EDS-mapping images of **2AggII**.

For monomers **3–4**, sigmoidal curves were obtained, reflective of the isodesmic mechanism. For the isodesmic model[3] adopted by **3–4**, the obtained fraction of aggregates (α) versus temperature was fitted according to Eq. (4):

$$\alpha(T) = \frac{1}{1+exp[\frac{T-T_m}{T^*}]} \qquad \text{Eq. (4)}$$

T_m is the temperature at which α is 0.5.

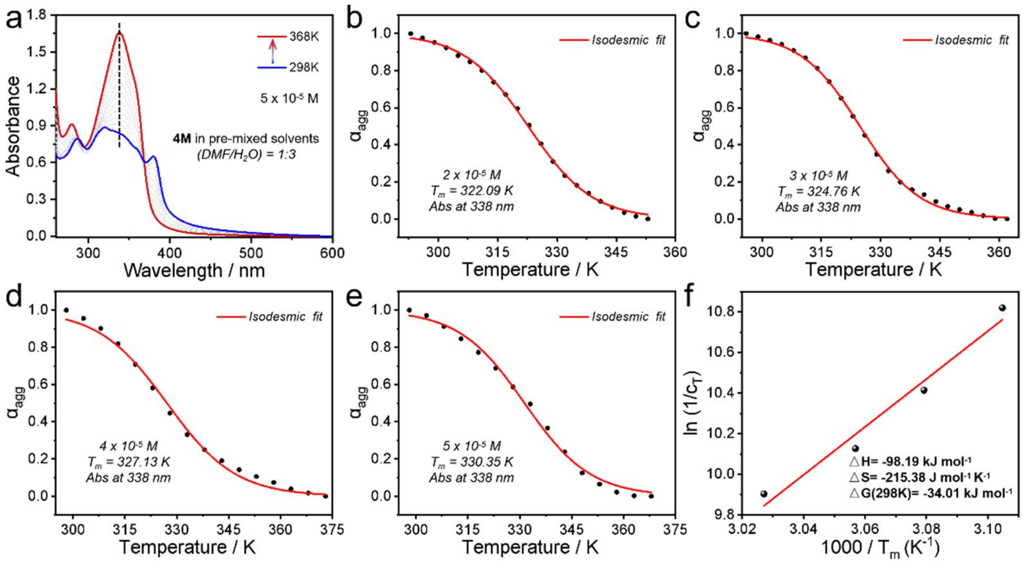

Supplementary Figure 19. (a) Temperature-dependent UV–Vis absorption of **4** in DMF/H_2O (1:3, *v*/*v*, $c = 5.00 \times 10^{-5}$ M) upon cooling (rate: 1 K/min) from 368 K to 298 K. (b-e) Plot of the variation

of the degree of aggregation monitored at λ = 338 nm versus temperature at different concentrations. The normalized melting curves at λ = 338 nm display sigmoidal curves, which are characteristic for the isodesmic assembling mechanism. (f) Van't Hoff plot of **4**. The red line denotes the respective linear fitting curve. According to the Van't Hoff plot, ΔG of the self-assembly process of **4** is calculated to be -34.01 kJ mol^{-1} at 298 K.

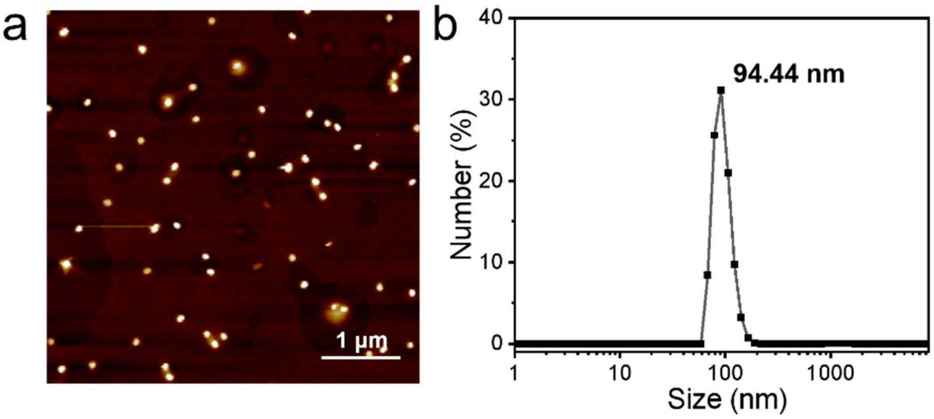

Supplementary Figure 20. (a) AFM image of **4**. (b) DLS results of **4** (5.00×10^{-5} M) in DMF/H_2O (1:3, *v*/*v*) at 298 K.

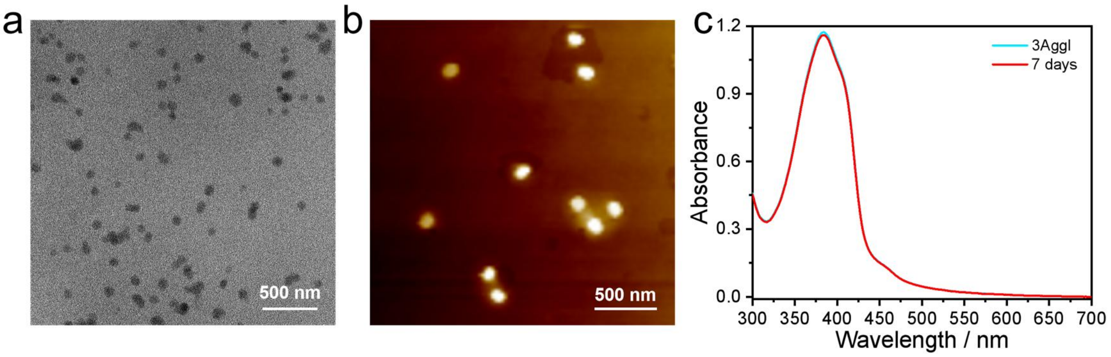

Supplementary Figure 21. (a) TEM, (b) AFM images and (c) time-dependent UV–Vis absorption spectra of **3AggI** in DMF/H_2O (1:3, *v*/*v*, $c = 5.00 \times 10^{-5}$ M) at 298 K.

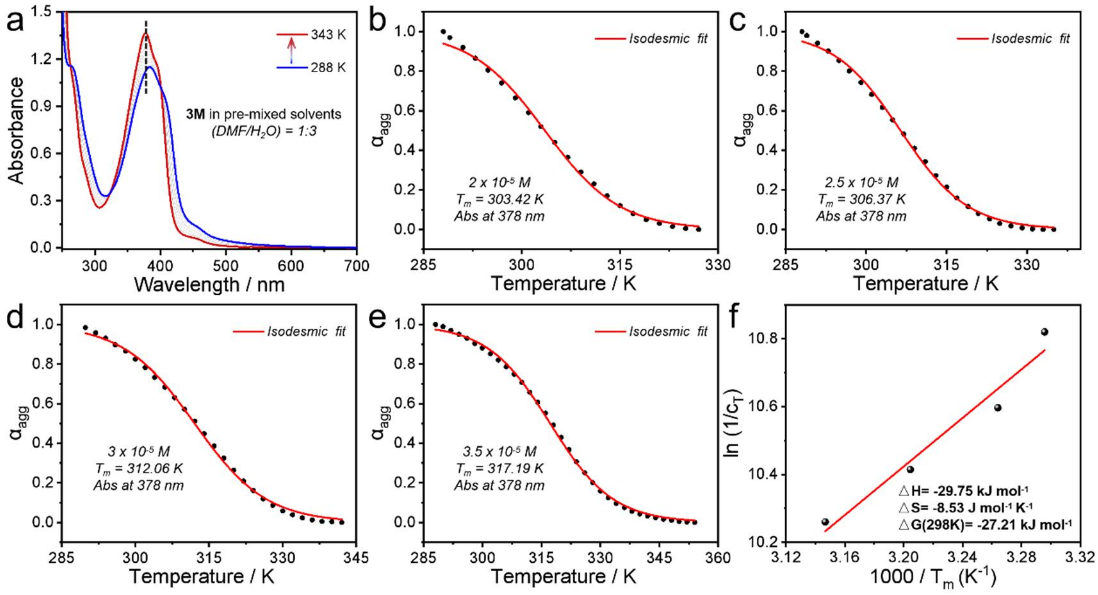

Supplementary Figure 22. Mechanism of self-assembly of **3AggI**. (a) Temperature-dependent UV–Vis absorption of **3AggI** in DMF/H_2O (1:3, *v*/*v*, *c* = 5.00 × 10^{-5} M) upon heating (rate: 1 K/min) from 288 K to 343 K. (b-e) Plot of the variation of the degree of aggregation monitored at λ = 378 nm versus temperature at different concentrations. The normalized melting curves at λ = 378 nm display sigmoidal curves, which are characteristic for the isodesmic assembling mechanism. (f) Van't Hoff plot of **3AggI**. The red line denotes the linear fitting curve. According to the Van't Hoff plot, ΔG of the self-assembly process of **3AggI** is calculated to be -27.21 kJ mol^{-1} at 298 K.

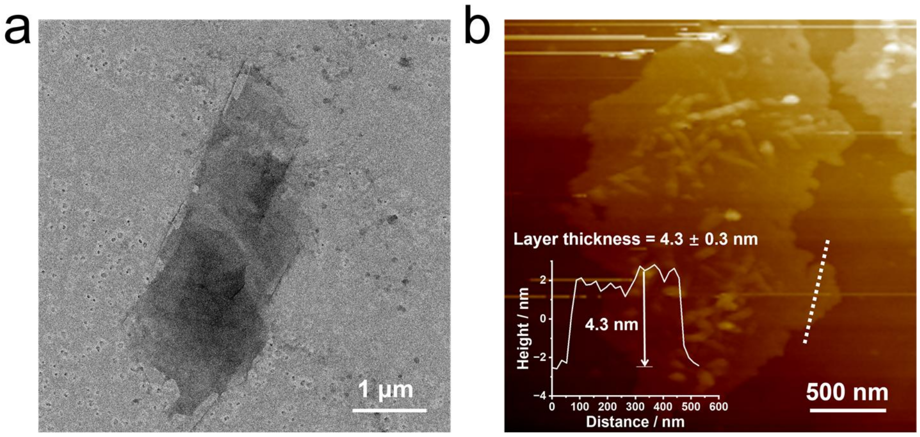

Supplementary Figure 23. (a) TEM and (b) AFM images of **3AggII** in DMF/H_2O (1:3, *v*/*v*, *c* = 5.00 × 10^{-5} M) at 298 K. Inset of (b) shows the height profile.

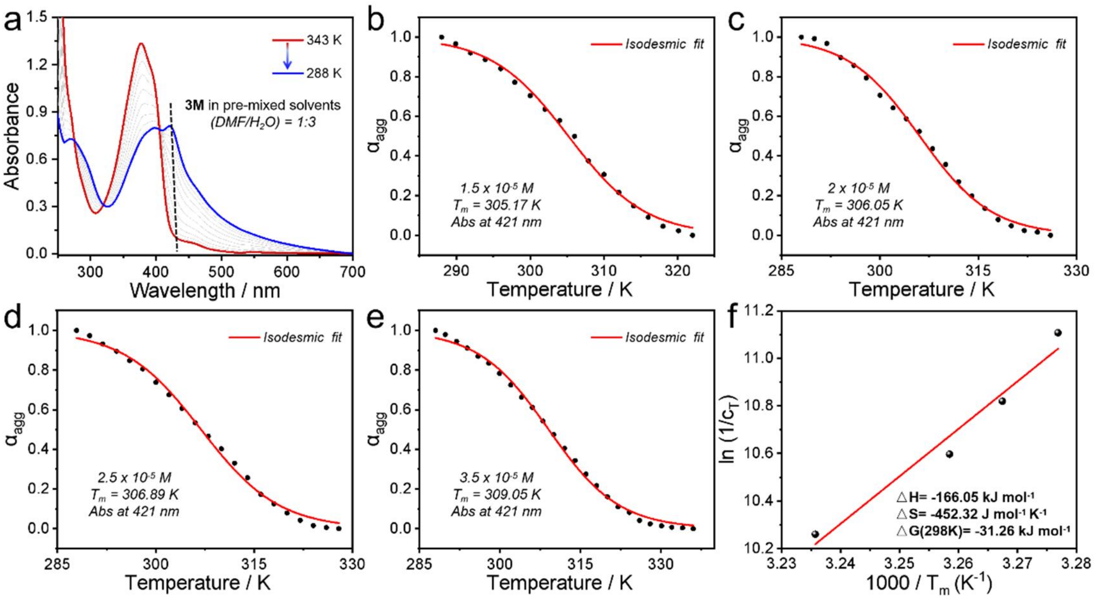

Supplementary Figure 24. Mechanism of self-assembly of **3AggII**. (a) Temperature-dependent UV–Vis absorption of **3AggII** in DMF/H_2O (1:3, *v*/*v*, $c = 5.00 \times 10^{-5}$ M) upon cooling (rate: 1 K/min) from 343 K to 288 K. (b–e) Plot of the variation of the degree of aggregation monitored at λ = 421 nm versus temperature at different concentrations. The normalized melting curves at λ = 421 nm display sigmoidal curves, which are characteristic for the isodesmic assembling mechanism. (f) Van't Hoff plot of **3AggII**. The red line denotes the respective linear fitting curve. According to the Van’t Hoff plot, ΔG of the self-assembly process of **3AggII** is calculated to be -31.26 kJ mol^{-1} at 298 K.

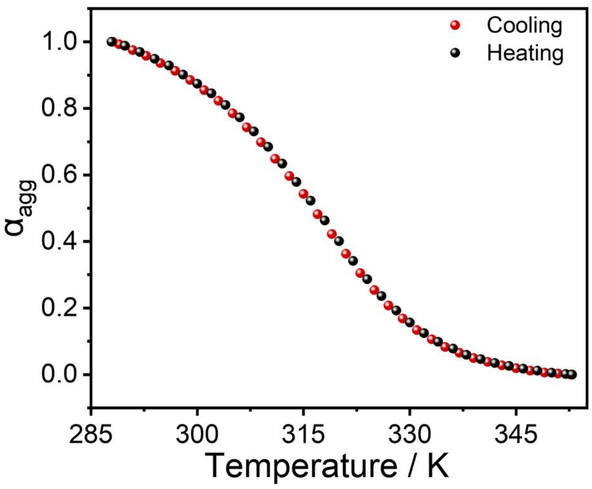

Supplementary Figure 25. Temperature-dependent degree of **3AggII** (α_{Agg}) calculated from the absorption intensities at λ = 421 nm observed in the heating and cooling processes.

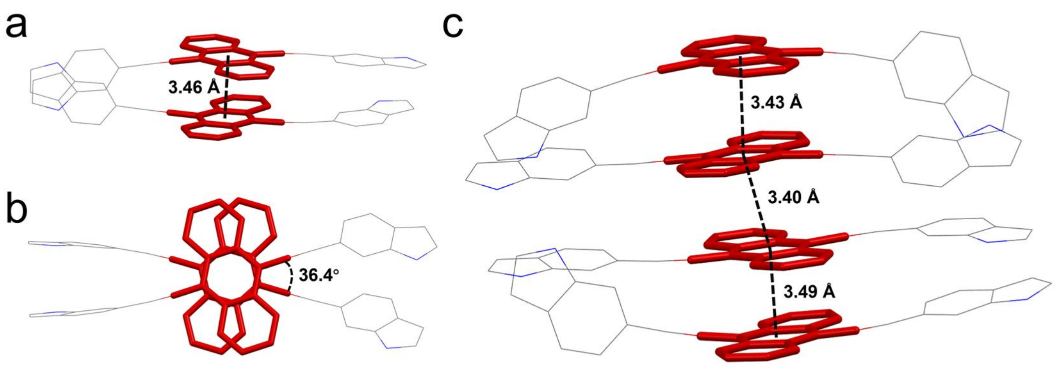

Supplementary Figure 26. Geometry-optimized structure of dimer (**1AggI$_2$**) at the (a) side view and (b) top view. (c) The side view of tetramer (**1AggI$_4$**) structures of **1AggI** in a face-to-face packing. The intermolecular π–π distance between the neighboring anthryl core was 3.46 Å and the torsional angle between the two neighboring anthryl rings was determined to be 36.4°. The intermolecular π–π distances between the neighboring anthryl core of **1AggI$_4$** were determined to be 3.40-3.49 Å (the averaged distances: 3.44 Å). For more visual observation, the Capped Sticks model is used for the central anthracene element, and the Wireframe model is used for the remaining atoms.

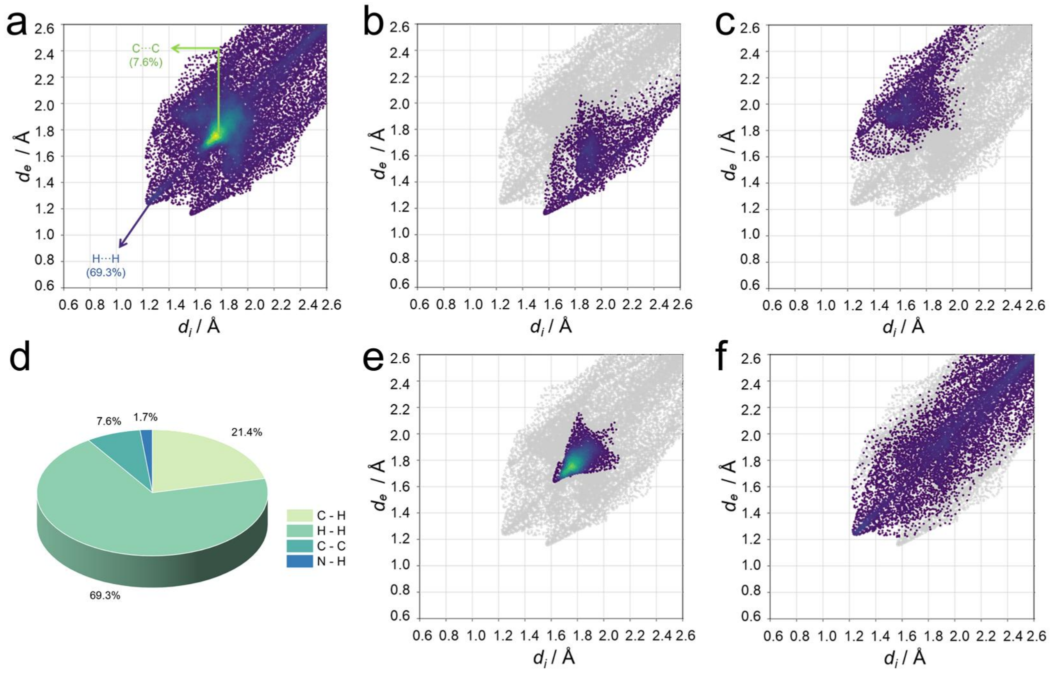

Supplementary Figure 27. (a) Short contact fraction distributions in **1AggI** dimer. Surface area of reciprocal contacts from **1AggI** dimer including (b) C···H; (c) H···C; (e) C···C; (f) H···H and (d) the corresponding pie chart.

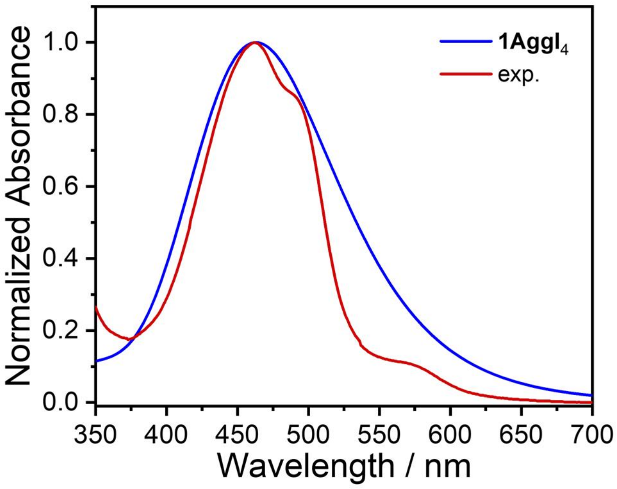

Supplementary Figure 28. The corresponding calculated electronic transition spectra of **1AggI$_4$** and experimental UV–Vis absorption spectra of **1AggI**. The spectrum of the calculated **1AggI$_4$** overlapped well with the experimental result, indicating the rationality of the optimized structure of **1AggI**.

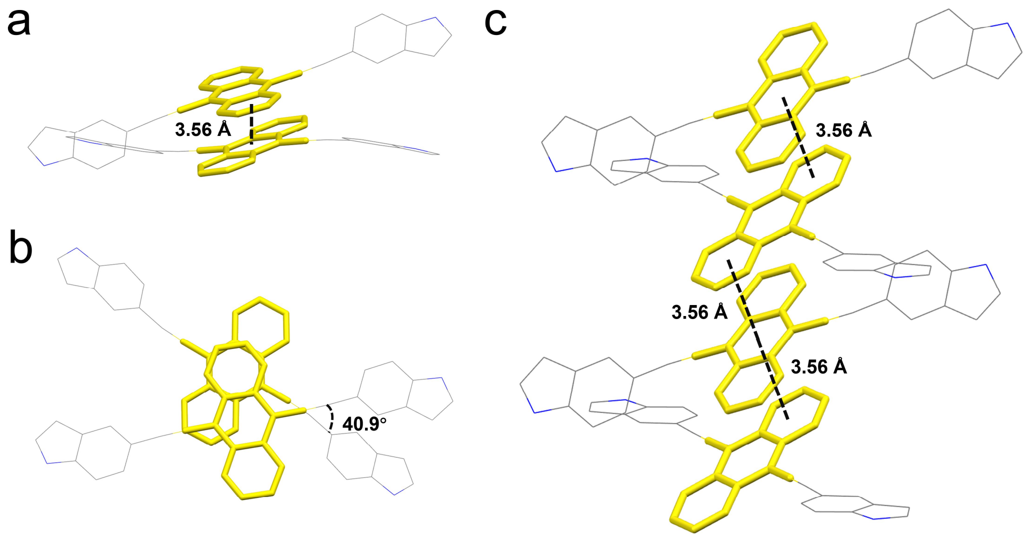

Supplementary Figure 29. Geometry-optimized structure of dimer (**1AggII$_2$**) at the (a) side view and (b) top view. (c) The side view of tetramer (**1AggII$_4$**) structures of **1AggII** in an offset packing. The intermolecular π–π distance between the central ring of anthryl core and the marginal ring of the adjacent anthryl were 3.56 Å and the torsional angles between the two neighboring molecular were determined to be 40.9°. For more visual observation, the Capped Sticks model is used for the central anthracene element, and the Wireframe model is used for the remaining atoms.

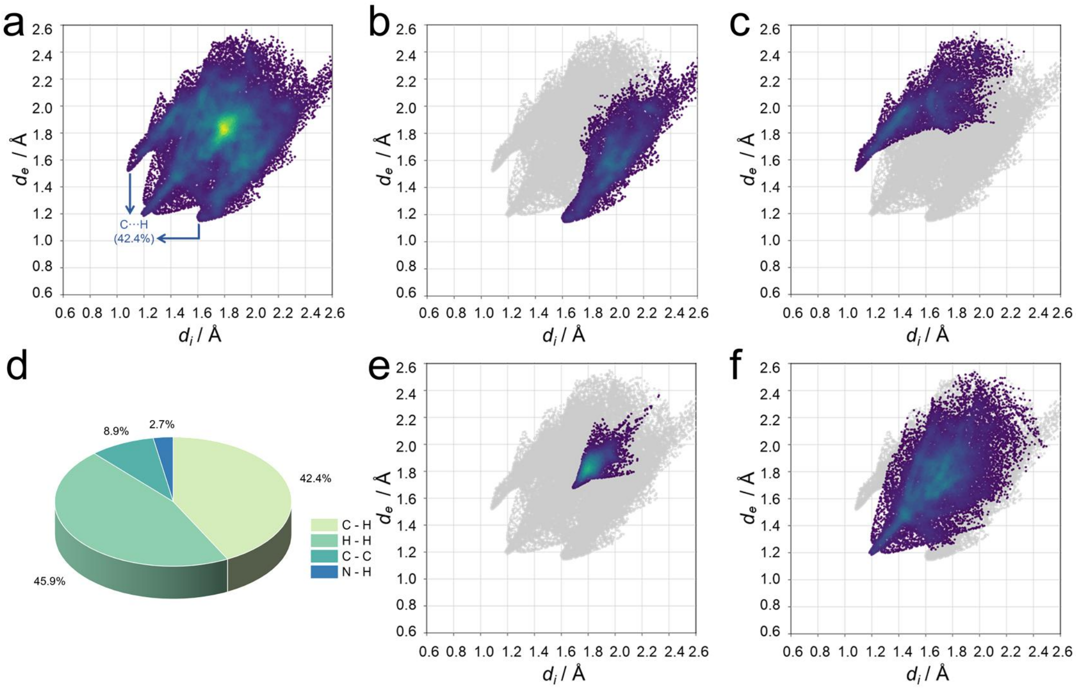

Supplementary Figure 30. (a) Short contact fraction distributions in **1AggII** dimer. Surface area of reciprocal contacts from **1AggII** dimer crystal including (b) C···H; (c) H···C; (e) C···C; (f) H···H and (d) the corresponding pie chart.

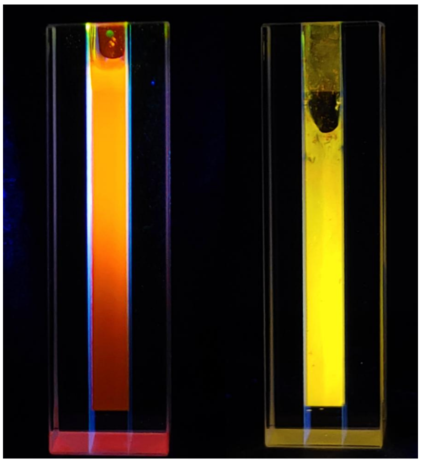

Supplementary Figure 31. Time-dependent photos of the corresponding luminescence under 365 nm UV light of **1** in the mixed solvent D_2O/DMSO-d_6 (3:7, *v*/*v*, 5.0 mM) at 298K: left, 0 min; right, 100 min. The varied emission of **1** in this condition was similar to that measured in the fluorescence spectra (Figure 2d in the main text), which agreed to obtain the identical metastable supramolecular polymerization results.

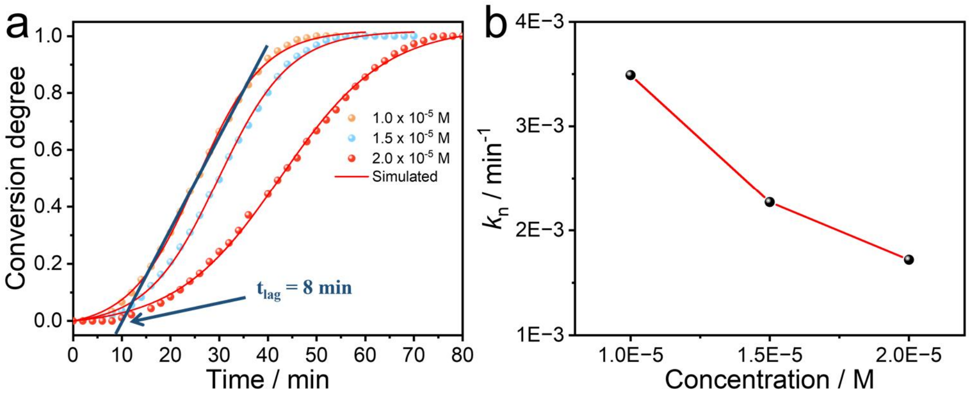

Supplementary Figure 32. (a) Fits of concentration-dependent kinetic data of **2** in DMF/H_2O (1:3, *v*/*v*) at 298 K to Finke–Watzky model. (b) Nucleation rate constant(k_n) versus concentration of **2**.

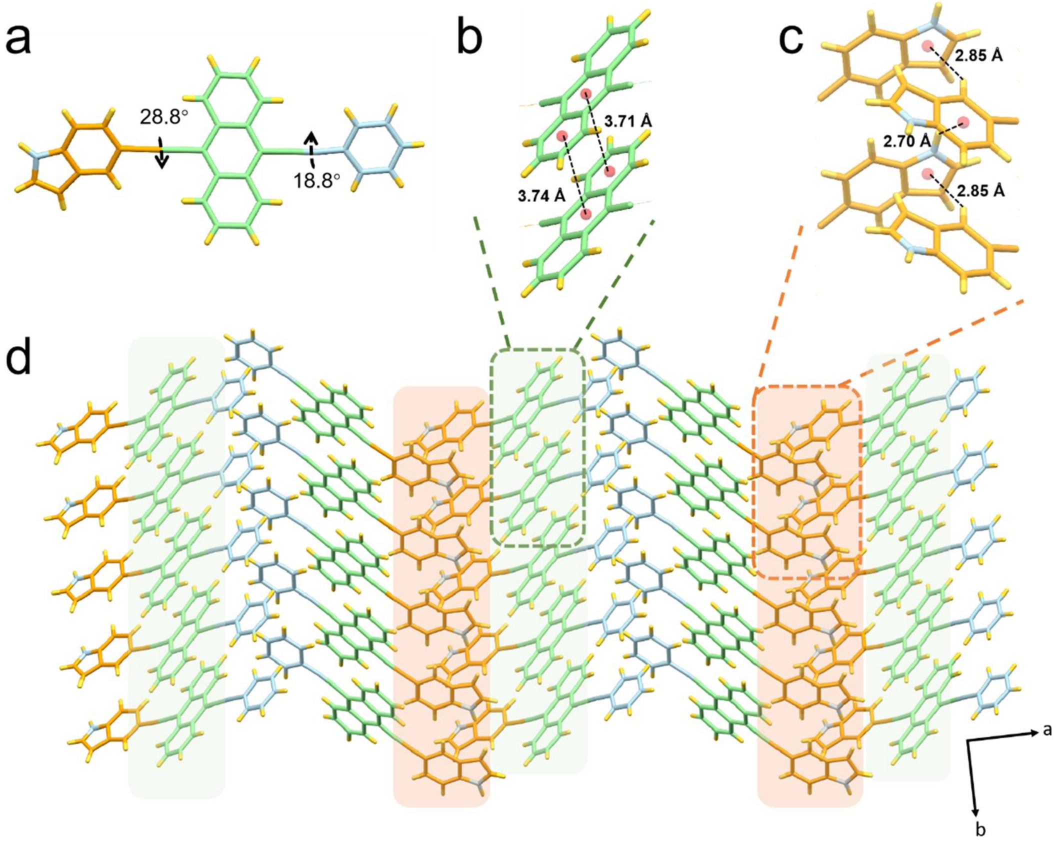

Supplementary Figure 33. Molecular stacking mode of the thermodynamically stable **2AggII**. (a)–(d) X-ray single crystal structures of **2AggII** (CCDC: 2351018) at different views. The intermolecular π-π interaction and C-H···π interaction are showed in (b) and (c), respectively. The orange and green colored background in (d) indicate the position of edge-to-face stacking of indoles and offset stacking of anthryl, respectively.

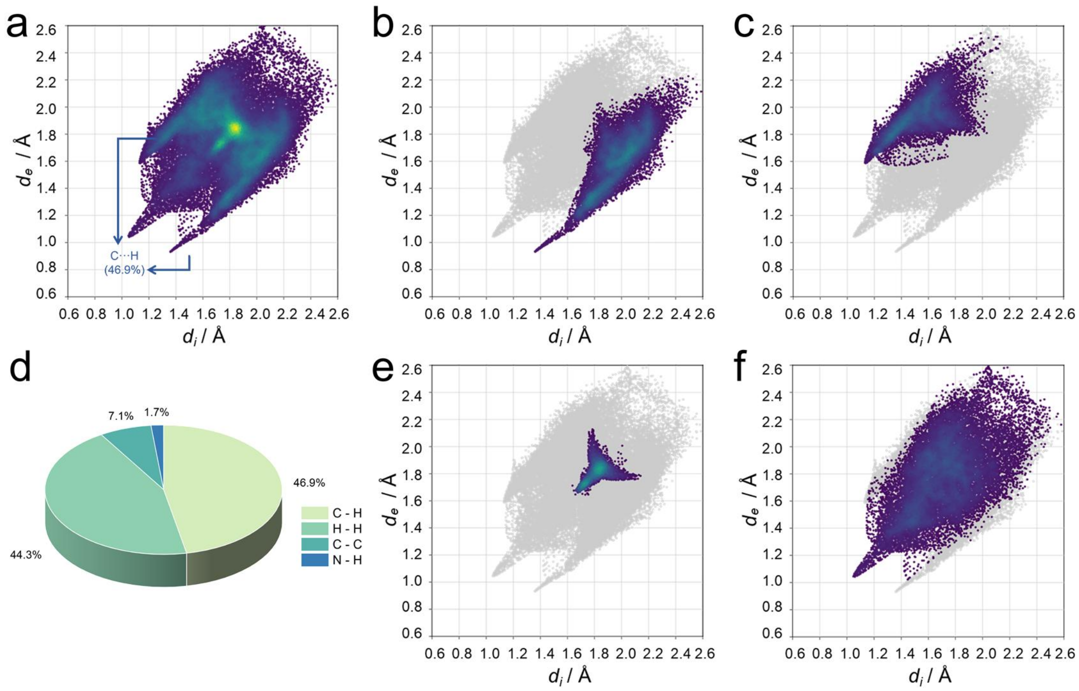

Supplementary Figure 34. (a) Short contact fraction distributions in **2AggII** dimer. Surface area of reciprocal contacts from **2AggII** dimer crystal including (b) C···H; (c) H···C; (e) C···C; (f) H···H and (d) the corresponding pie chart.

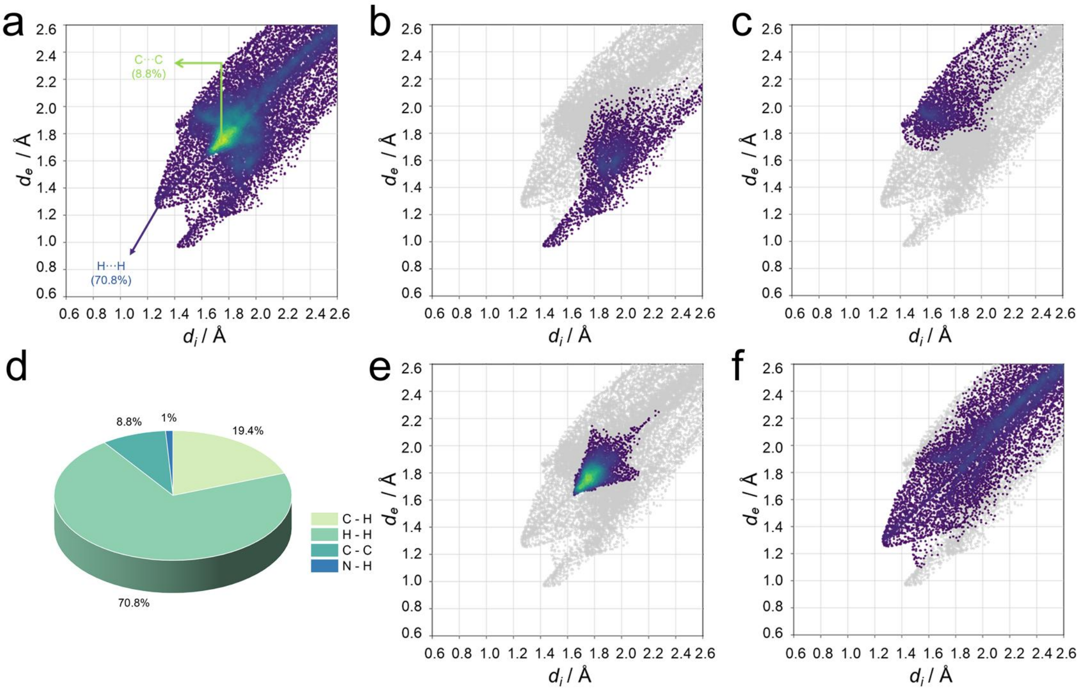

Supplementary Figure 35. (a) Short contact fraction distributions in **2AggI** dimer. Surface area of reciprocal contacts from **2AggI** dimer including (b) C···H; (c) H···C; (e) C···C; (f) H···H and (d) the corresponding pie chart.

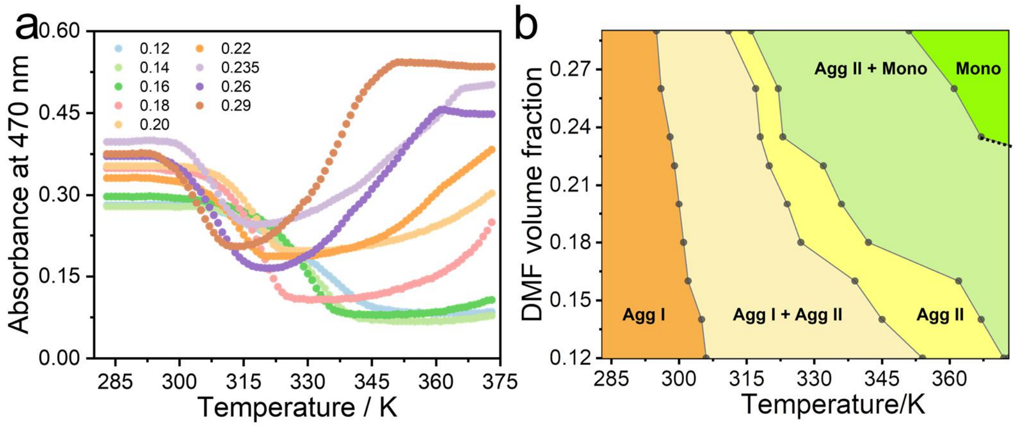

Supplementary Figure 36. (a) Temperature-dependent absorption spectra of **2** (c = 2.00×10^{-5} M) separately monitored at 470 nm in H_2O/DMF mixtures with different DMF volume fraction ranging from 1% to 31%. (b) State diagram of the predominance of the respective species of **2** in dependency of temperature and DMF volume fraction in DMF/H_2O mixture. The temperatures for the transformation of **2AggI**-to-**2AggII**-to-**2M** were determined and plotted as a function of the solvent composition, displaying the precise state diagram. The boundary between adjacent states is not sharp (the mixture states of **2AggI**+**2AggII** and **2AggII**+**2M**), ascribing to the consecutive transformation.

Supplementary Table 6. Critical temperature obtained from the temperature-dependent denaturation experiments of **2** in H_2O/DMF mixtures with different DMF volume fraction.

DMF volume fraction	T_{AggI} (K)	$T_{\mathrm{AggI\text{-}AggII}}$ (K)	$T_{\mathrm{AggII\text{-}Mono}}$ (K)	T_{Mono} (K)
0.12	306	354	372	
0.14	305	345	367	
0.16	302	339	362	
0.18	301	327	342	
0.2	300	324	336	
0.22	299	320	332	
0.235	298	318	323	367
0.26	296	317	322	361
0.29	295	311	316	351

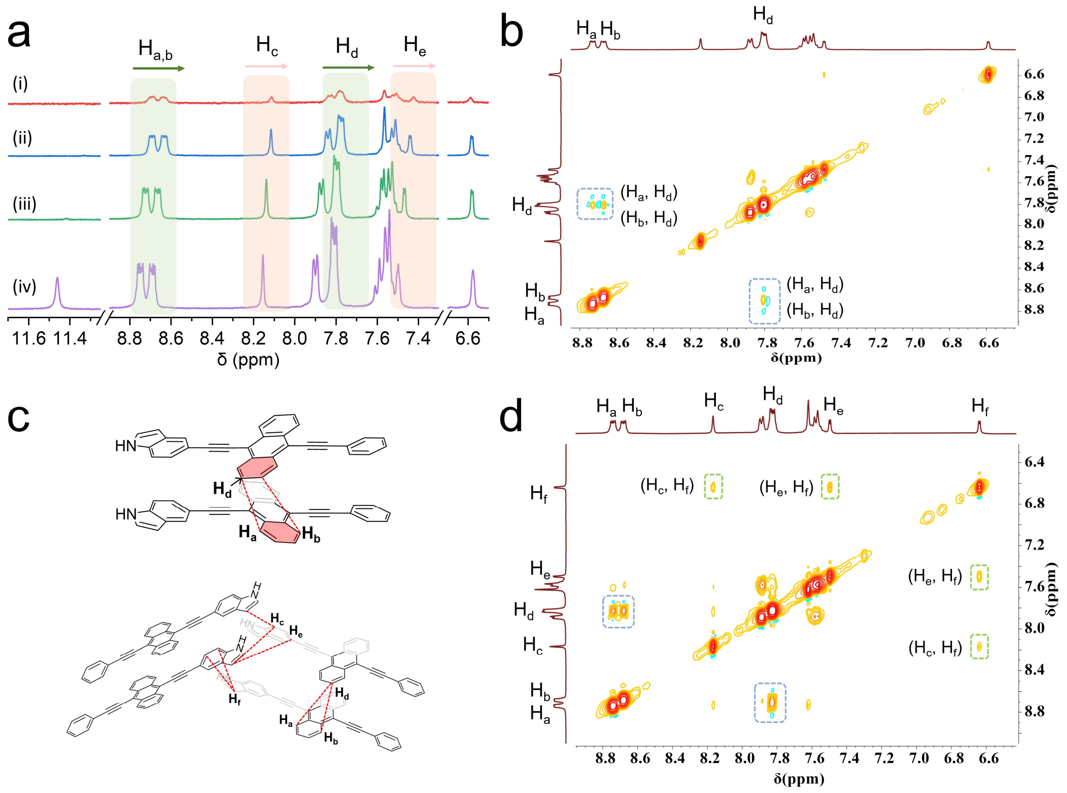

Supplementary Figure 37. Analysis of the non-covalent interactions for **2AggI** and **2AggII** by ^{1}H NMR measurements. (a) Time-dependent partial ^{1}H NMR (400 MHz, 298 K, 5.00 mM, D_2O/DMSO-d_6, 3:7, *v*/*v*) spectral variations of **2AggI**: (i) 0 min, (ii) 10 min, (iii) 20 min and (iv) 30min. Partial ^{1}H-^{1}H NOESY NMR (400 MHz, 298 K, 5.00 mM, D_2O/DMSO-d_6, 3:7, *v*/*v*) spectra of (b) **2AggI** and (d) **2AggII**, and (c) the corresponding correlated protons positions of each dimers. The well-defined sharp signals of aromatic protons on the fresh prepared **2AggI** gradually shifted upfield, broadened and submerged in the baseline. This strong shielding effect signified the transformation from small oligomers to large-sized aggregates. The protons correlation was then verified by 2D NOESY. Apparent NOE correlated signals were observed in (H_a, H_d) and (H_b, H_d) on the marginal anthryl ring, reflective of a strong π-π interaction of anthryl in a face-to-face manner. In comparison, apart from the similar correlations, the NOE signals of (H_c, H_f) and (H_e, H_f) representing multifold C-H···π interactions in indole units appeared for **2AggII**.

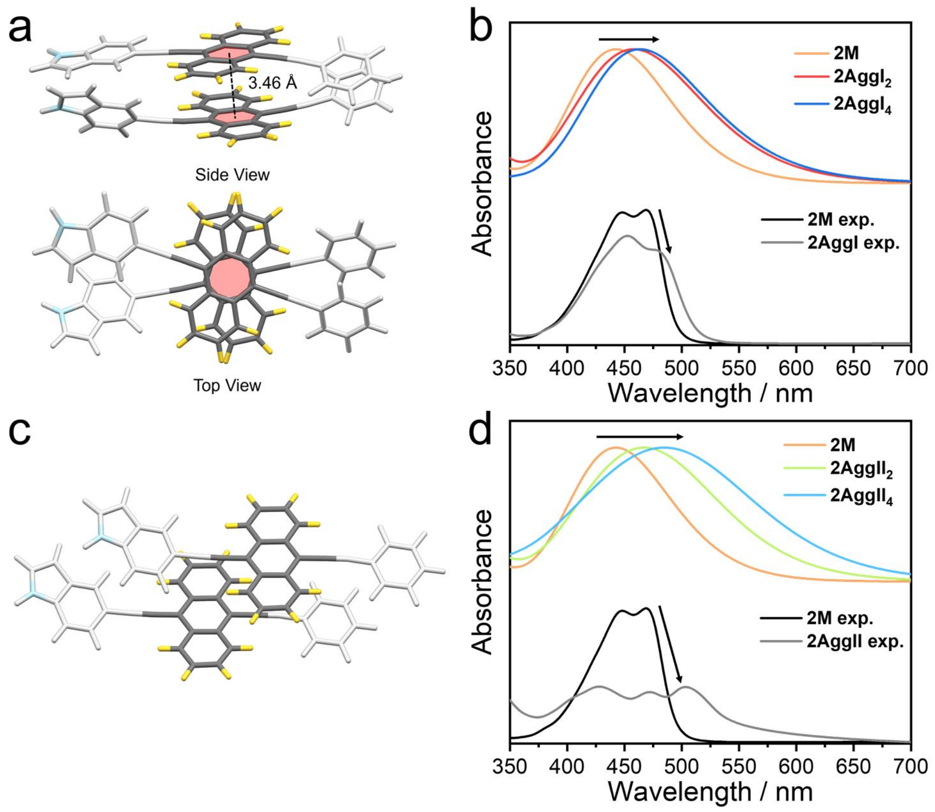

Supplementary Figure 38. The optimized dimer structures of (a) **2AggI** and (c) **2AggII** at the side view and top view, together with the corresponding calculated and experimental electronic transition spectra of (b) **2AggI** (including monomer **2M**, dimer **2AggI$_2$**, and tetramer **2AggI$_4$**) and (d) **2AggII** (including monomer **2M**, dimer **2AggII$_2$**, and tetramer **2AggII$_4$**). The simulated absorption spectra are obtained from TD-DFT computation in water solvent. The arrows in (b) and (d) represent the spectral variation trends. The optimized metastable **2AggI$_2$** and **2AggI$_4$** exhibited a face-to-face stacking, in which the central ring of anthryl core was parallel to the adjacent anthryl central ring with a π–π distance of 3.46 Å. To verify the rationality of the optimized structure of **2AggI**, the resulted spectroscopic signatures were compared. The calculated electronic transition spectra of **2AggI$_2$** and **2AggI$_4$** displayed a sequential red-shift with respect to the monomer absorption, which is consistent with the experimentally measured trends. For **2AggII**, DFT calculations for its dimers and tetramers successfully reproduced both aggregations compared to the single crystal results. The more obvious spectral red-shift was observed compared to that of **2AggI**, agreeing well with the measured results.

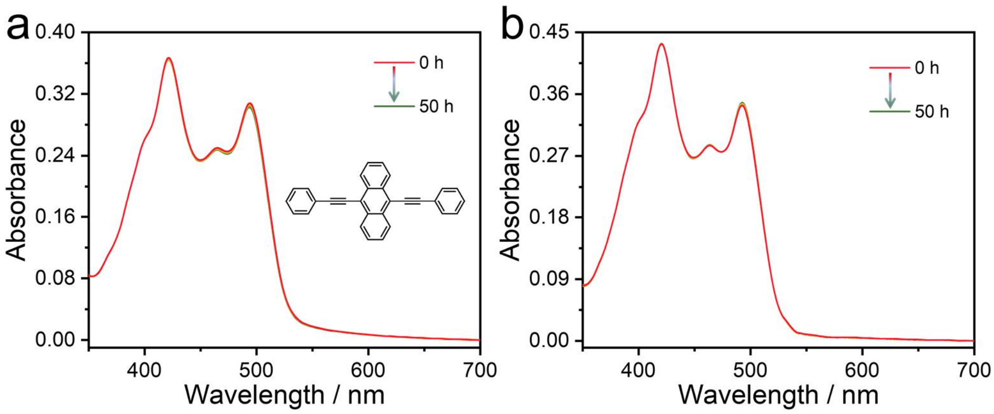

Supplementary Figure 39. Time-dependent UV–Vis absorption of 9,10-bis(phenylethynyl)anthracene (c = 3.00 × 10^{-5} M) in DMF/H_2O at 298 K. (a) DMF/H_2O = 1:24, *v*/*v*; (b) DMF/H_2O = 1:3, *v*/*v*. Inset of (a) shows the chemical structure of 9,10-bis(phenylethynyl)anthracene.

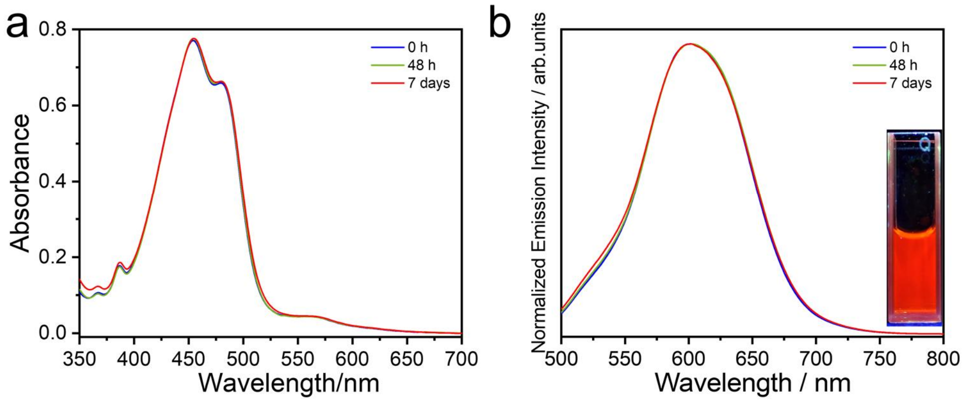

Supplementary Figure 40. Time-dependent (a) UV–Vis absorption and (b) fluorescence emission spectra of **5AggI** in DMF/H_2O (1:3, *v*/*v*, c = 3.00 × 10^{-5} M) at 298 K. Inset of (b) is the photograph of **5AggI** under UV light.

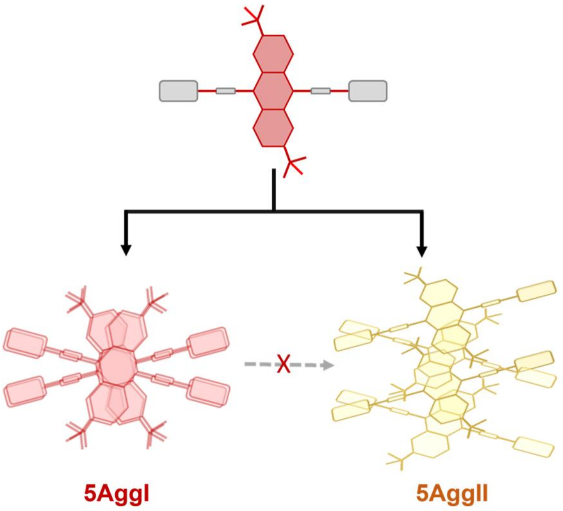

Supplementary Figure 41. Schematic representation of supramolecular polymerization behaviors of monomer **5**. In the self-assembly structures of **5**, the face-to-face packing model is shown in red, while slipped packing model is shown in yellow.

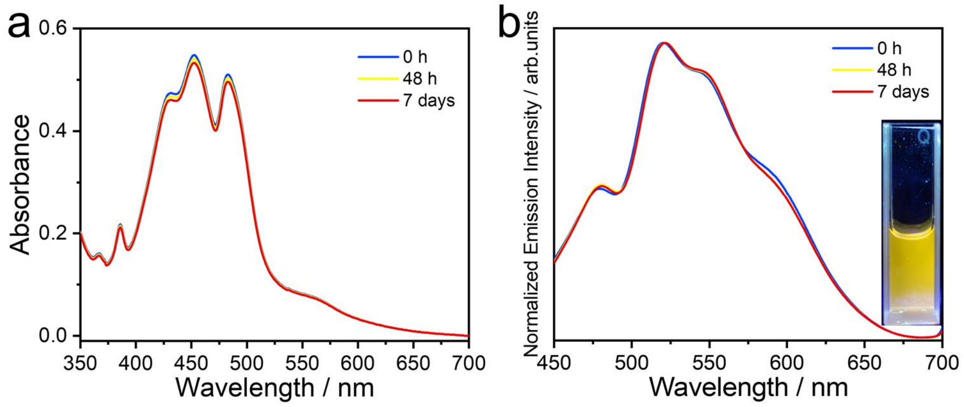

Supplementary Figure 42. Time-dependent (a) UV–Vis absorption and (b) fluorescence emission spectra of **5AggI** in DMF/H_2O (1:3, *v*/*v*, $c = 3.00 \times 10^{-5}$ M) at 298 K. **5AggII** is obtained by heating **5AggI** to 373 K and then cooling to room temperature. Inset of (b) is the photograph of **5AggII** under UV light. a. u. represents arbitrary unit.

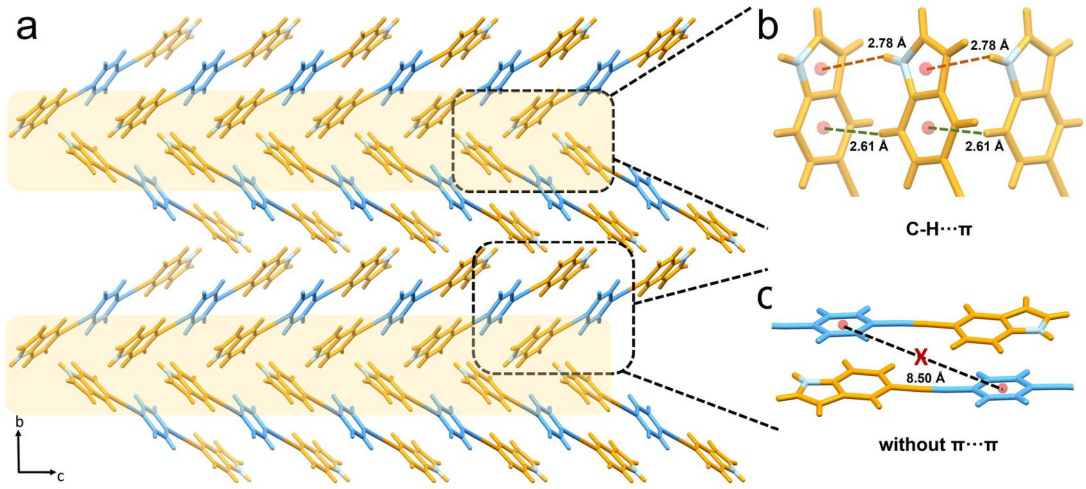

Supplementary Figure 43. Molecular stacking mode of the thermodynamically stable **4**. (a)–(c) X-ray single crystal structures of **4** (CCDC: 2368743). The orange colored background indicates the position of edge-to-face stacking of indoles. Multifold C-H···π interactions resulted from the edge-to-face stacking of indoles were observed for the aggregated **4**, showing the distances of 2.61 and 2.78 Å. However, the central phenyl was parallelly arranged with the adjacent ones, exhibiting no offset stacking with a distance of 8.50 Å. Therefore, due to the offset stacking of phenyl could be ignored when the aromatic core is as small as phenyl, **4** is mainly driven by the C-H···π interactions resulted from the edge-to-face stacking of indoles.

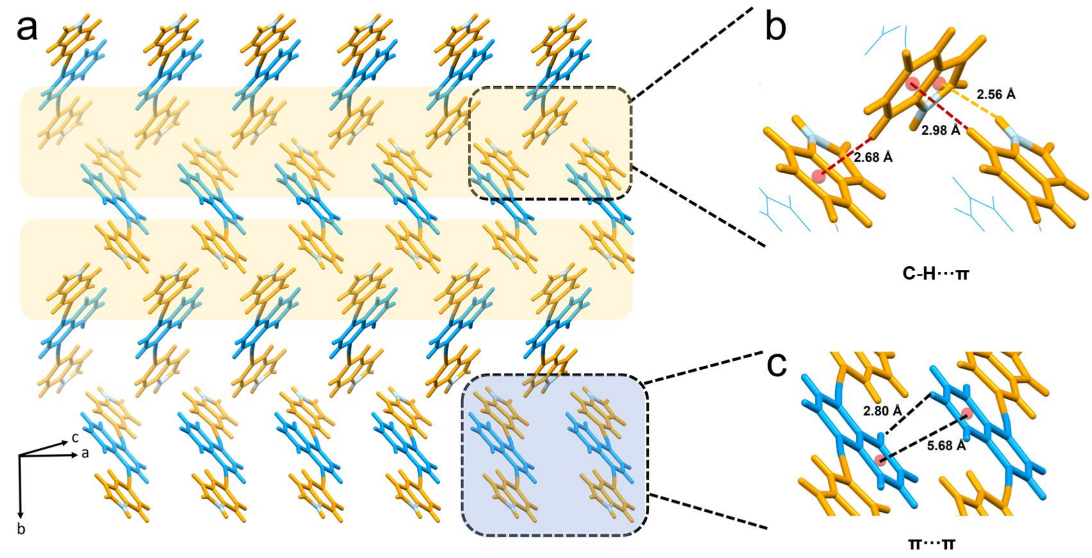

Supplementary Figure 44. Molecular stacking mode of the thermodynamically stable **3AggII**. (a)–(c) X-ray single crystal structures of **3AggII** (CCDC: 2368744) at different views. The C-H···π is

showed in (b), respectively. The orange colored background in (a) indicate the position of edge-to-face stacking of indoles. Multifold C-H···π interactions resulted from the edge-to-face stacking of indoles were observed for **3AggII**, showing the distances of 2.56–2.98 Å. Although the offset stacking of the central naphthyl on **3** is weak, this stacking could no longer be neglected compared to the offset stacking of phenyl on **4** (5.68 Å versus 8.50 Å). Even so, the multifold C-H···π interactions still dominate, resulting in the two stable supramolecular polymers driving by edge-to-face stacking of indoles assisted with offset stacking of naphthyl (see below).

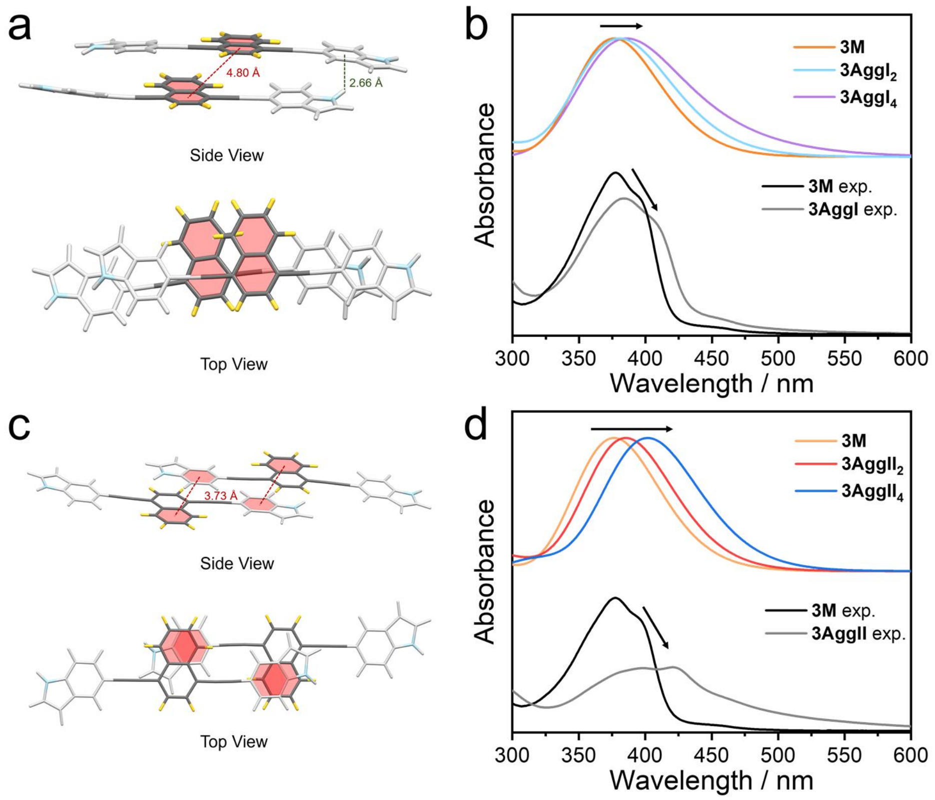

Supplementary Figure 45. The optimized dimer structures of (a) **3AggI** and (c) **3AggII** at the side view and top view, together with the corresponding calculated and experimental electronic transition spectra of (b) **3AggI** (including monomer **3M**, dimer **3AggI$_2$**, and tetramer **3AggI$_4$**) and (d) **3AggII** (including monomer **3M**, dimer **3AggII$_2$**, and tetramer **3AggII$_4$**). The simulated absorption spectra are obtained from TD-DFT computation in water solvent. The arrows in (b) and (d) represent the spectral variation trends. The single crystal structures have well demonstrated the stacking mode of **3AggII**. However, we were unable to obtain single crystal for **3AggI**. To get insights into the

stacking mode of **3AggI**, spectroscopic experiments and quantum chemical calculations were employed. In detail, geometries of monomers, dimers (**3AggI$_2$**), and tetramers (**3AggI$_4$**) of **3** were built and optimized via DFT calculations by a B3LYP/6-31G(d) basis set, and the corresponding electronic transition spectra were simulated by TD-DFT calculations at the same computational level. The optimized metastable **3AggI$_2$** exhibited an offset stacking of the central naphthyl with a distance of 4.80 Å, and a C-H···π interactions of 2.66 Å resulted from the edge-to-face stacking of indoles (Supplementary Figure 45a). Driven by the offset stacking of naphthyl and edge-to-face stacking of indoles, **3AggI** is very stable upon long-time standing at room temperature. To verify the rationality of the optimized structure of **3AggI**, the resulted spectroscopic signals were compared (Supplementary Figure 45b). The calculated electronic transition spectra of **3AggI$_2$** and **3AggI$_4$** displayed a sequential red-shift with respect to the monomer absorption, which is consistent with the experimentally measured trends (**3M** exp. versus **3AggI** exp.). Besides, for **3AggII**, DFT calculations (the same computational basis set of **3AggI**) for its dimers successfully reproduced both aggregations compared to the single crystal results (Supplementary Figure 45c). A greater degree of red-shift with respect to the monomer absorption was observed compared to that of **3AggI**, agreeing well with the measured results (Supplementary Figure 45d).

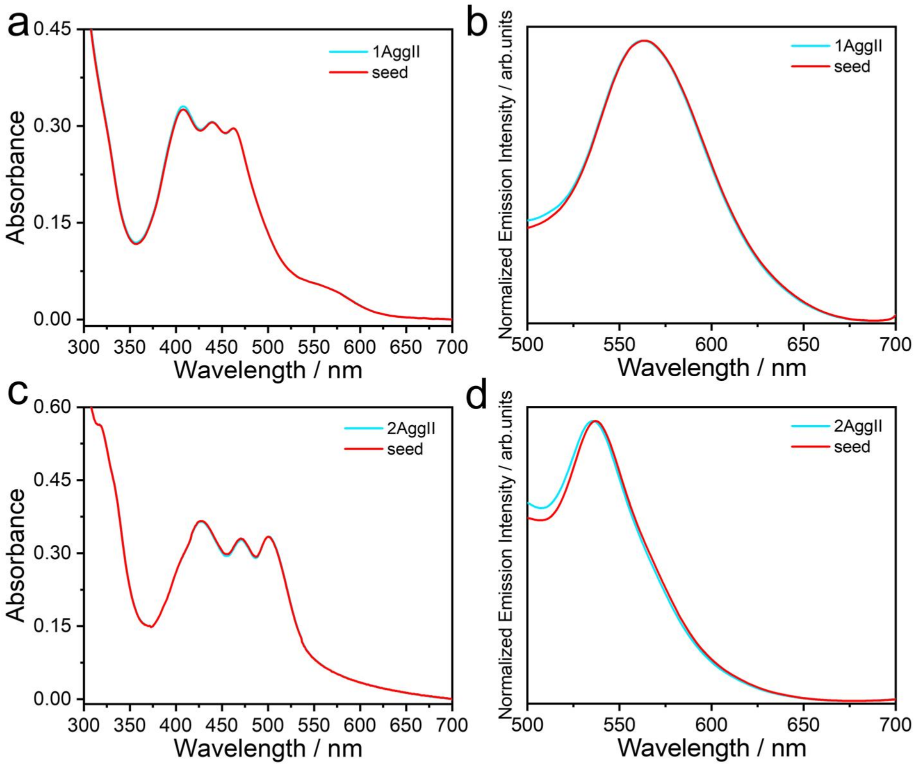

Supplementary Figure 46. (a) and (c) UV–Vis absorption and (b) and (d) fluorescence emission spectra of ultrasonic seeds **1** (DMF/H_2O, 1:3, *v*/*v*, $c = 2.00 \times 10^{-5}$ M) and **2** (DMF/H_2O, 1:3, *v*/*v*, $c = 2.00 \times 10^{-5}$ M) and the corresponding supramolecular polymers. It can be concluded that ultra-sonication hardly changed the optical properties.

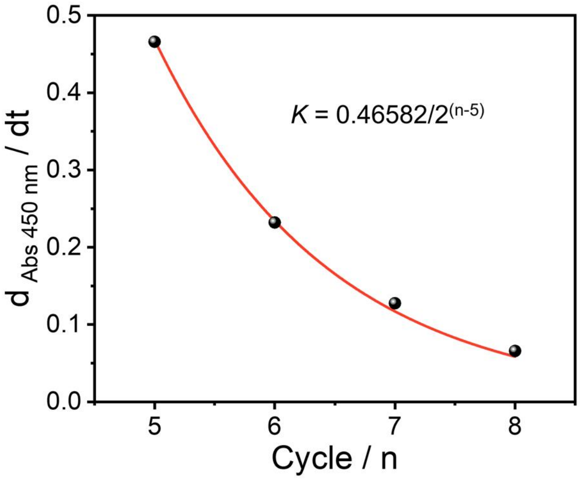

Supplementary Figure 47. Initial polymerization rate ($d_{Abs\ 450\ nm}/dt$) for 5−8 cycles as a function of the cycle number n (n ≥ 5) and the non-linear fitting curve. The polymerization rate decays exponentially with a base of 2^{-1}.

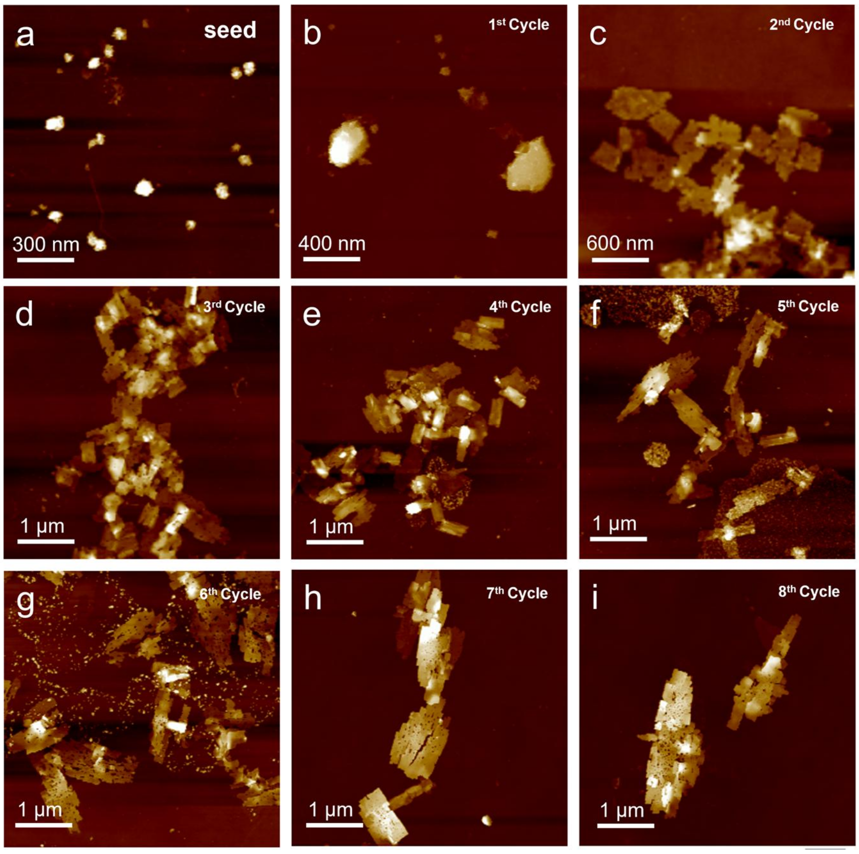

Supplementary Figure 48. AFM images of (a) **2seed** and (b)–(i) the gradually enlarged aggregates of **2AggII** obtained after the seeded supramolecular polymerization.

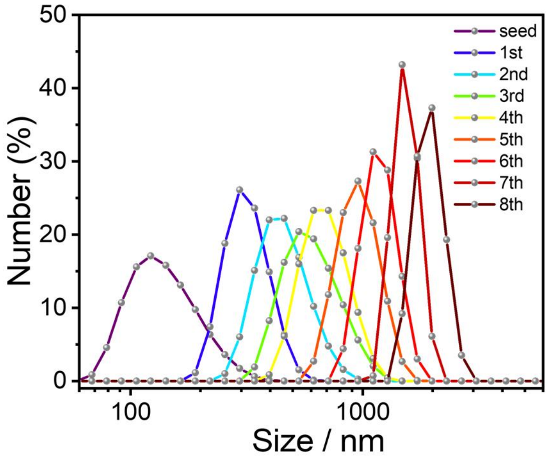

Supplementary Figure 49. DLS results of **2seed** and the gradually enlarged aggregates of **2AggII** obtained after the seeded supramolecular polymerization.

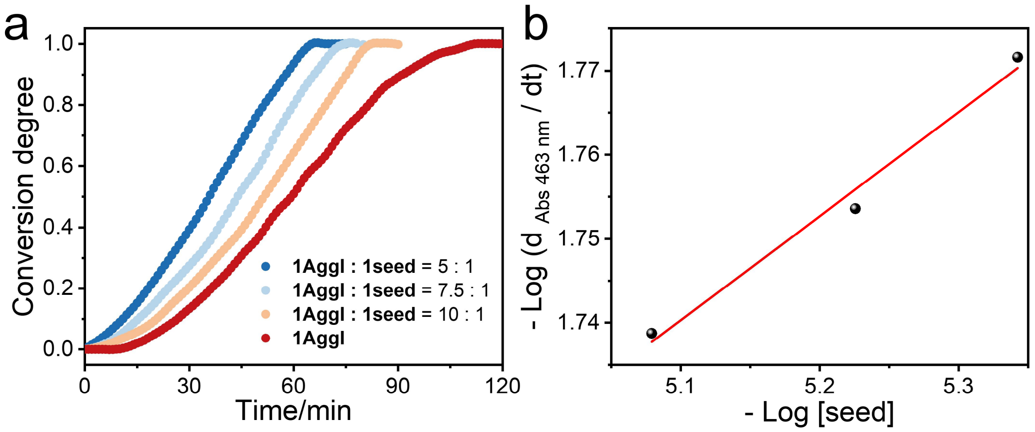

Supplementary Figure 50. (a) Time course of seeded supramolecular polymerization of **1** monitored as changes in absorbance intensities at λ = 463 nm initiated by addition of **1seed** to a H_2O/DMF (24:1, *v*/*v*, $c = 4.00\times10^{-5}$ M, 293 K) solution of **1AggI** in the molar ratios of 1:5, 1:7.5 and 1:10. (b) Log-log plot of the rate of polymerization as a function of the concentration of 1seed. The correlation coefficient of solid line linear fitting was 0.992.

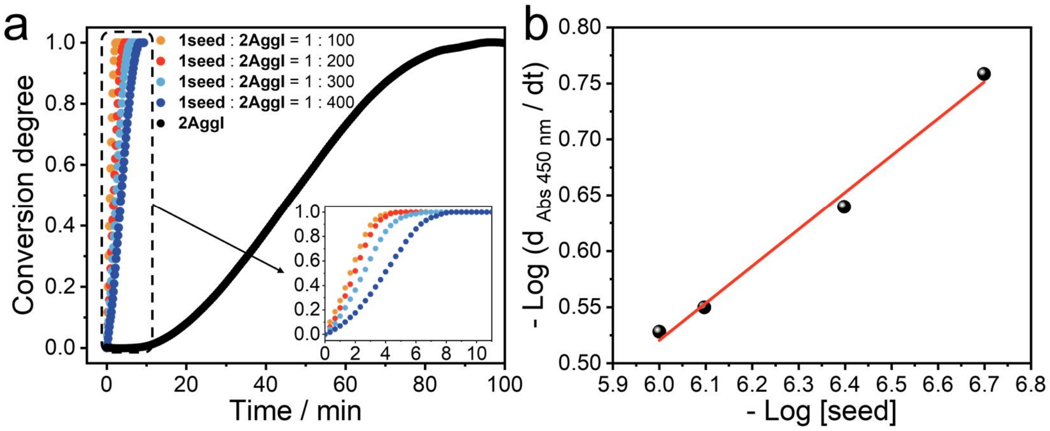

Supplementary Figure 51. (a) Time course of seeded supramolecular polymerization of **2** monitored as changes in absorbance intensities at λ = 450 nm initiated by addition of **1seed** to a H_2O/DMF (24:1, *v*/*v*, $c = 2.00\times10^{-5}$ M) solution of **2AggI** in the molar ratios of 1:100, 1:200, 1:300 and 1:400. Inset shows the partial amplified area. (b) Log-log plot of the rate of polymerization as a function of the concentration of **1seed**. The correlation coefficient of solid line linear fitting was 0.998.

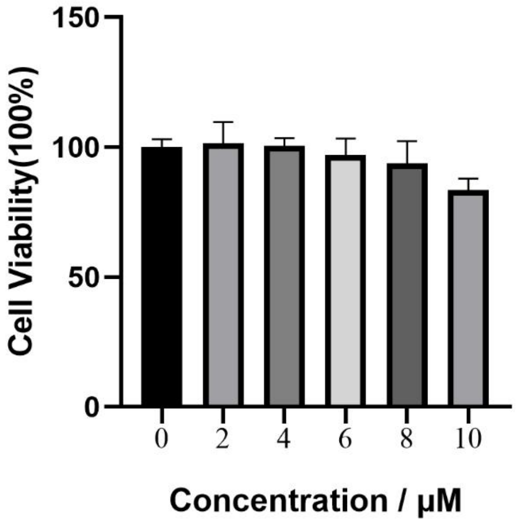

Supplementary Figure 52. Cytotoxicity tests on A549 cells at different concentrations (0, 2, 4, 6, 8, 10 μM) for **1** after 24 h incubation. The cell viability was assessed via MTT assay. The values presented are the mean ± SD (n=3).

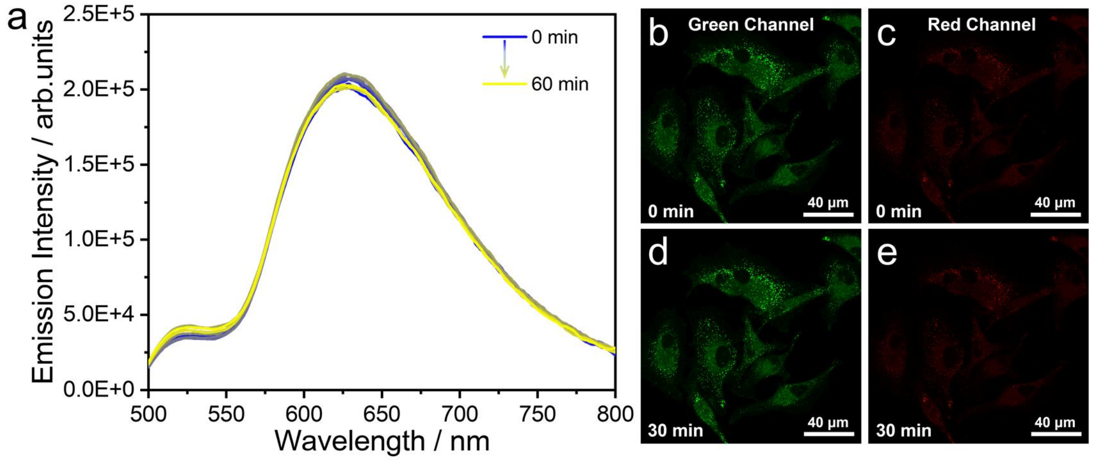

Supplementary Figure 53. (a) Time-dependent fluorescence emission spectra of **5** (c = 0.80×10^{-5} M) in DMEM-H complete medium with 0.05% DMSO. CLSM images of A549 cells co-stained with **5** for (b)–(c) 0 min and (d)–(e) 30 min at green channel and red channel, respectively. Green channel: λ_{ex} = 405 nm, λ_{em} = 500-599 nm. Red channel: λ_{ex} = 405 nm, λ_{em} = 600-700 nm.

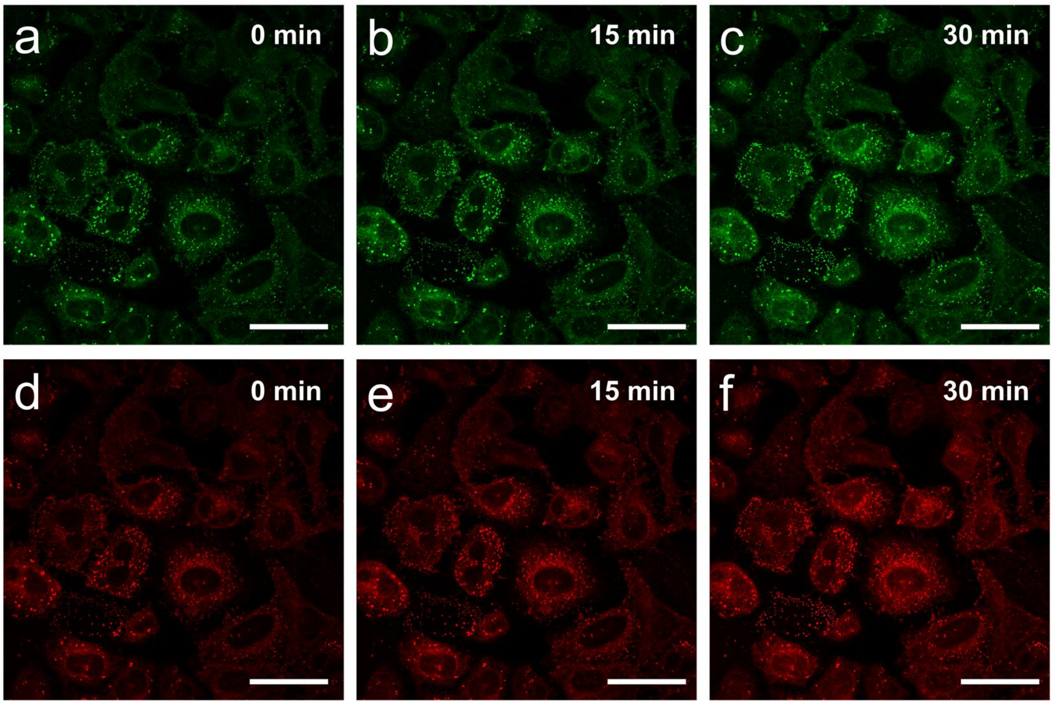

Supplementary Figure 54. CLSM images of the spontaneous transformation of **1AggI** to **1AggII** in B16-F10 cells at (a)–(c) green channel (λ_{ex} = 405 nm, λ_{em} = 500–599 nm) and (d)–(f) red channel (λ_{ex} = 405 nm, λ_{em} = 600–700 nm) for 0, 15, and 30 min. Scale bar: 40 μm.

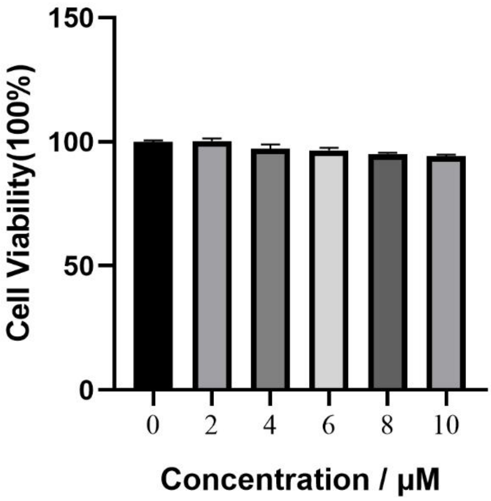

Supplementary Figure 55. Cytotoxicity tests on B16-F10 cells at different concentrations (0, 2, 4, 6, 8, 10 μM) for **1** after 24 h incubation. The cell viability was assessed via MTT assay. The values presented are the mean ± SD (n=3).

3. Synthetic routes to the targeted monomers 1-5

3.1 Materials

Commercial solvents and reagents were used as received without further purification. All analytical reagents of TEA, DMF, MeCN, THF, ethyl acetate and toluene were purchased from Guangdong Guanghua Sci-Tech Co., Ltd., China. 5-i-1H-indole (98%), 9,10-dibromoanthracene (98.5%) and 1,4-dibromonaphthalene (98%) were purchased from Bide Pharmatech Co., Ltd., China. CuI (99%), $Pd(PPh_3)_4$ (99%), $Pd(PPh_3)_2Cl_2$ (99%), tetrabutylammonium fluoride (98%) were purchased commercially from Energy Chemical.

3.2 Synthesis of monomers **1-2**

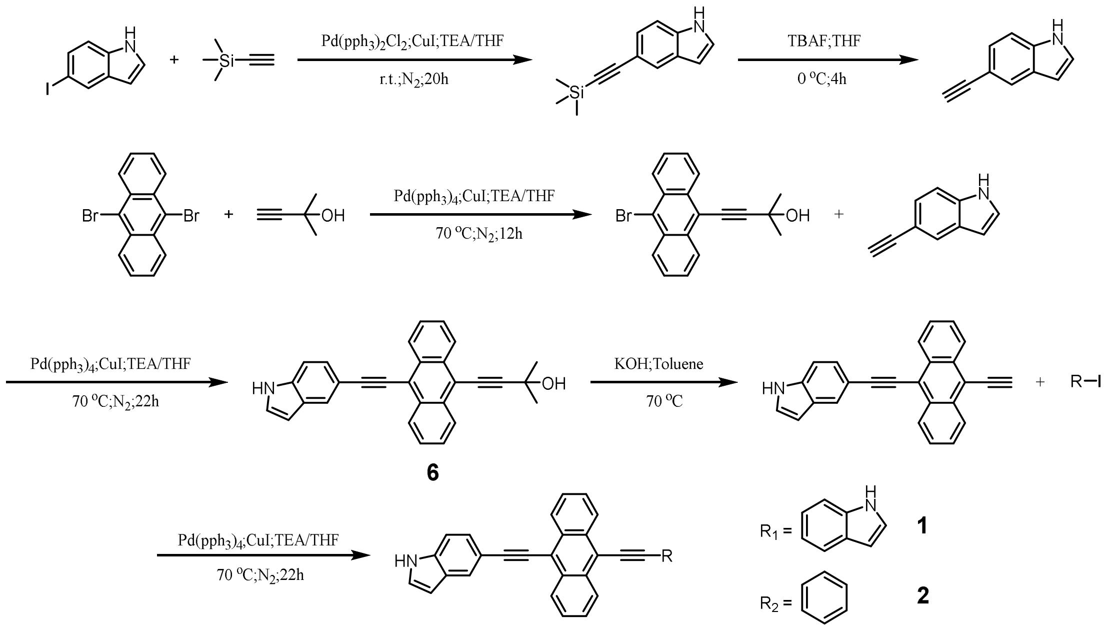

Supplementary Figure 56. Synthesis routes toward monomers **1-2**.

5-((Trimethylsilyl)ethynyl)-1H-indole. CuI (45.2 mg, 0.24 mmol, 0.04 equiv), $Pd(PPh_3)_2Cl_2$ (84.3 mg, 0.12 mmol, 0.02 equiv) and 5-I-1H-indole (1506.2 mg, 6.2 mmol, 1.0 equiv) were first added into a Schlenk tube. TEA (5 mL) and DMF (15 mL) were then added under a nitrogen atmosphere. After that, ethynyltrimethylsilane (914.3 mg, 1.5 mL, 9.3 mmol, 1.5 equiv) were added. The reaction mixture was stirred at room temperature for 20 h. After cooling to room temperature, the mixture was quenched with 30 mL water. Then the solvent was removed under reduced pressure, the crude product was purified by silica-gel column chromatography using mixed solvents of petroleum ether/ethyl acetate (20:1, *v*/*v*) as the eluent to give a desired product in yellow oil (1040.8

mg, yield 80%). ^{1}H NMR (400 MHz, $CDCl_3$): δ (ppm) = 8.18 (s, 1H), 7.80 (s, 1H), 7.29 (s, 2H), 7.19 (t, 1H), 6.51- 6.50 (m, 1H), 0.25 (s, 9H).

5-Ethynyl-1H-indole. The product of the previous step (1000 mg, 4.8mmol, 1.0 equiv) was dissolved in THF (20 mL), then tetrabutylammonium fluoride (1840.5 mg, 1.9 mL, 7.1 mmol, 1.5 equiv) was added to the solution, and the reaction was carried out at 0°C for 4 hours. After back to room temperature, the mixture was quenched with 30 mL water. Then the solvent was removed under reduced pressure, the crude product was purified by silica-gel column chromatography using mixed solvents of petroleum ether/dichloromethane (4:1, *v*/*v*) as the eluent to give a desired product in black oil (614.2 mg, yield 90%). ^{1}H NMR (400 MHz, $CDCl_3$): δ (ppm) = 8.27 (br, 1H), 7.89 (s, 1H), 7.38 (s, 2H), 7.28-7.26 (m, 1H), 6.60-6.59 (m, 1H), 3.06 (s, 1H).

4-(10-bromoanthracen-9-yl)-2-methylbut-3-yn-2-ol. To the mixture of 9,10-dibromoanthracene (3.0 g, 9mmol, 1.0 equiv), CuI (11.4 mg, 0.06 mmol, 0.01 equiv), and $Pd(PPh_3)_4$ (207.9 mg, 0.18mmol, 0.02 equiv) were added THF (20 mL) and TEA (20 mL) under a nitrogen atmosphere. While stirring, 2-methyl-3-butyn-2-ol (1.5 g, 6 mmol, 2.0 equiv) in THF (5 mL) was injected through a syringe. The reaction mixture was stirred at 70 °C overnight under a nitrogen atmosphere. Upon completion, the solution was evaporated in vacuo to dryness. the crude product was purified by silica-gel column chromatography using mixed solvents of petroleum ether/dichloromethane (4:1, *v*/*v*) as the eluent to give a desired product in orange solid (1518.1 mg, yield 50%). ^{1}H NMR (400 MHz, $CDCl_3$) δ (ppm) = 8.64−8.45 (m, 4H),7.67−7.57 (m, 4H), 1.85 (s, 6H).

Synthesis of compound 6. To the mixture of 4-(10-bromoanthracen-9-yl)-2-methylbut-3-yn-2-ol (1.0 g, 3mmol, 1.0 equiv), 5-Ethynyl-1H-indole (846.0 mg, 6 mmol, 2.0 equiv), CuI (11.4 mg, 0.06 mmol, 0.02 equiv.), and $Pd(PPh_3)_4$ (34.7 mg, 0.03mmol, 0.01 equiv) were added THF (20 mL) and TEA (20 mL) under nitrogen atmosphere. The reaction mixture was stirred at 70 °C overnight under a nitrogen atmosphere. Upon completion, the solution was evaporated in vacuo to dryness. The crude product was purified by silica-gel column chromatography using mixed solvents of petroleum ether/dichloromethane (1:1, *v*/*v*) as the eluent to give a desired product in orange solid (523.2 mg, yield 44%). ^{1}H NMR (400 MHz, $CDCl_3$) δ (ppm) = 8.76 (d, J = 8.6 Hz, 2H), 8.54 (d, J = 8.4 Hz, 2H), 8.32 (s, 1H), 8.12 (s, 1H), 7.68–7.57 (m, 5H), 7.47 (d, J = 8.3 Hz, 1H), 7.29 (s, 1H),

6.64 (s, 1H), 1.86 (s, 6H). ^{13}C NMR (100 MHz, DMSO-d_6) δ (ppm) = 136.10, 131.33, 130.96, 127.82, 127.41, 126.88, 126.73, 124.56, 124.35, 118.17, 116.92, 112.28, 112.13, 109.41, 105.30, 101.61, 83.38, 76.84, 64.32, 40.13, 39.92, 39.71, 39.50, 39.29, 39.08, 38.87, 31.72. ESI-MS (m/z): $[M]^+$ calcd 399.1618, found 399.1610.

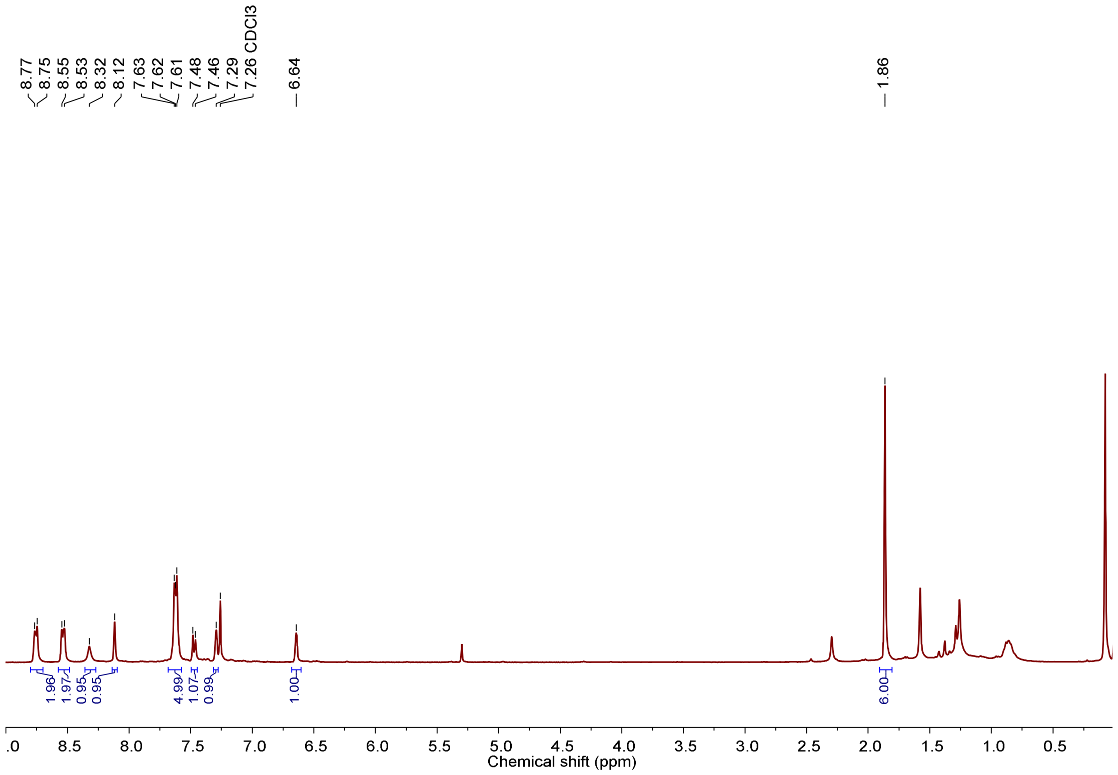

Supplementary Figure 57. ^{1}H NMR spectrum (400 MHz, $CDCl_3$, 298 K) of compound **6**.

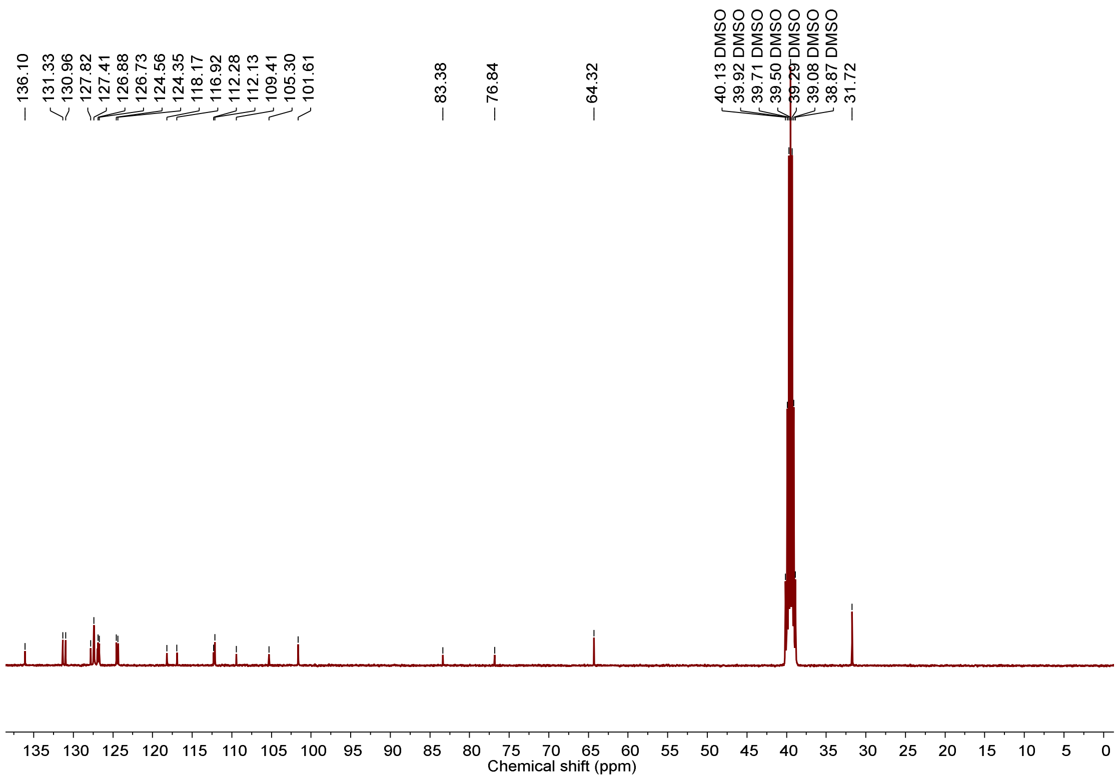

Supplementary Figure 58. ^{13}C NMR spectrum (100 MHz, DMSO-d_6, 298 K) of compound **6**.

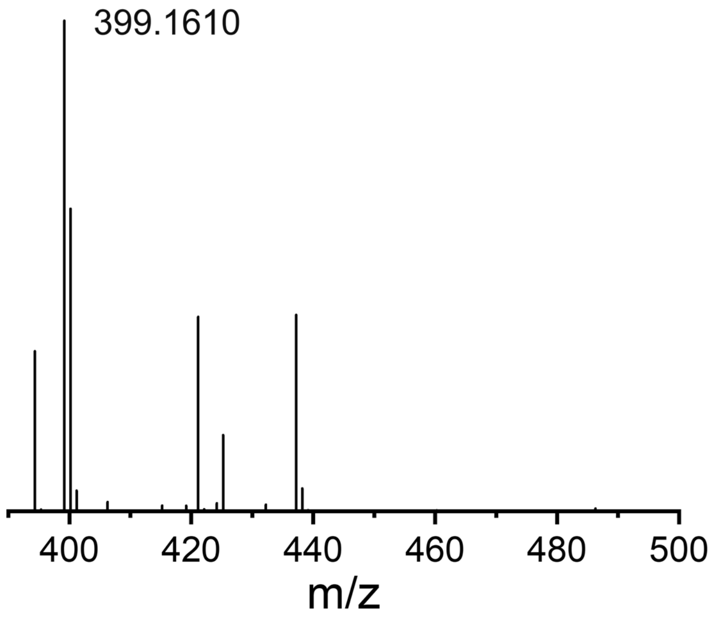

Supplementary Figure 59. HR-ESI-MS spectrum of compound **6**.

5-((10-ethynylanthracen-9-yl)ethynyl)-1H-indole. To a toluene (30 mL) solution of **6** (500 mg, 1.8mmol) was added KOH (0.22 g, 9 mmol), and the mixture was stirred at 90 °C for over 50 min and monitored by TLC. Upon completion, the solution was evaporated in vacuo to dryness. The crude product was purified by silica-gel column chromatography using mixed solvents of petroleum ether/dichloromethane (4:1, *v*/*v*) as the eluent to give a desired product in orange solid (162.8 mg, yield 38%). Because the product was easy to deteriorate, no further characterization was carried out.

Synthesis of monomer 1. To the mixture of 5-((10-ethynylanthracen-9-yl)ethynyl)-1H-indole (150 mg, 0.44 mmol, 1.0 equiv), 5-I-1H-indole (213.8 mg, 0.88 mmol, 2.0 equiv), CuI (1.9 mg, 0.01 mmol, 0.02 equiv), and $Pd(PPh_3)_4$ (11.6 mg, 0.01mmol, 0.01 equiv) were added THF (20 mL) and TEA (20 mL) under nitrogen atmosphere. The reaction mixture was stirred at 70 °C overnight under a nitrogen atmosphere. Upon completion, the solution was evaporated in vacuo to dryness. The crude product was purified by silica-gel column chromatography using mixed solvents of petroleum ether/dichloromethane (3:2, *v*/*v*) as the eluent to give a desired product in red solid (142.2 mg, yield 71%). ^{1}H NMR (400 MHz, DMSO-d_6) δ (ppm) = 11.44 (s, 1H), 8.74 (dd, J = 6.6, 3.2 Hz, 2H), 8.15 (s, 1H), 7.81 (dd, J = 6.7, 3.2 Hz, 2H), 7.62 – 7.54 (m, 2H), 7.51 – 7.47 (m, 1H), 6.58 (s, 1H). ^{13}C NMR (100 MHz, DMSO-d_6) δ (ppm) =136.09, 131.11, 127.84, 127.39, 126.94, 124.58, 124.33, 117.68, 112.40, 112.14, 105.30, 101.62, 83.62. ESI-MS (m/z): $[M]^+$ calcd 456.1621, found 456.1617.

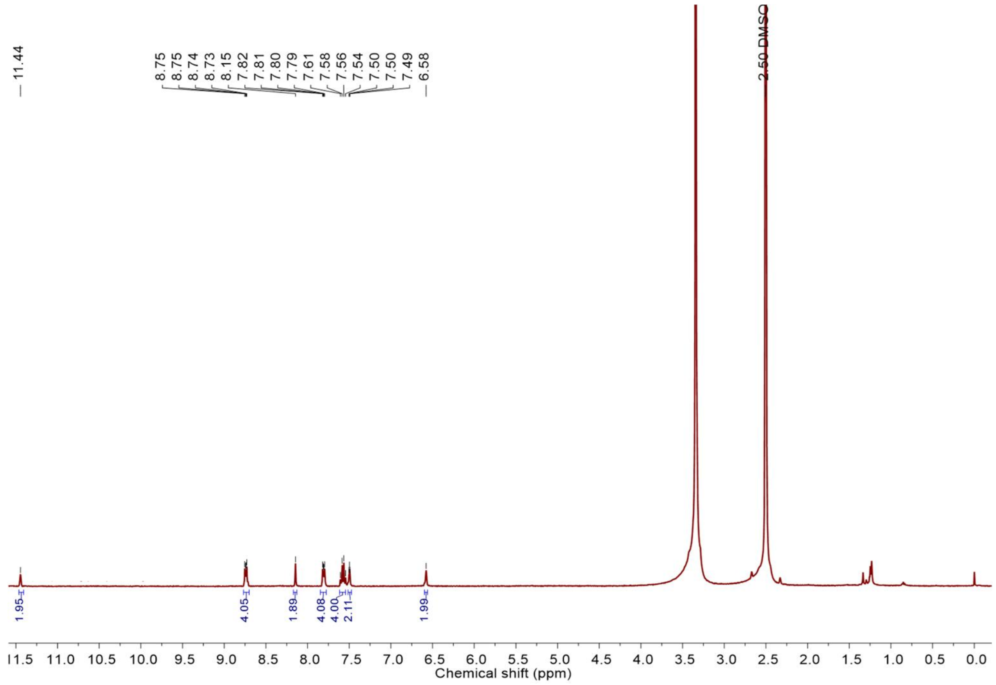

Supplementary Figure 60. ^{1}H NMR spectrum (400 MHz, DMSO-d_6, 298 K) of monomer **1**.

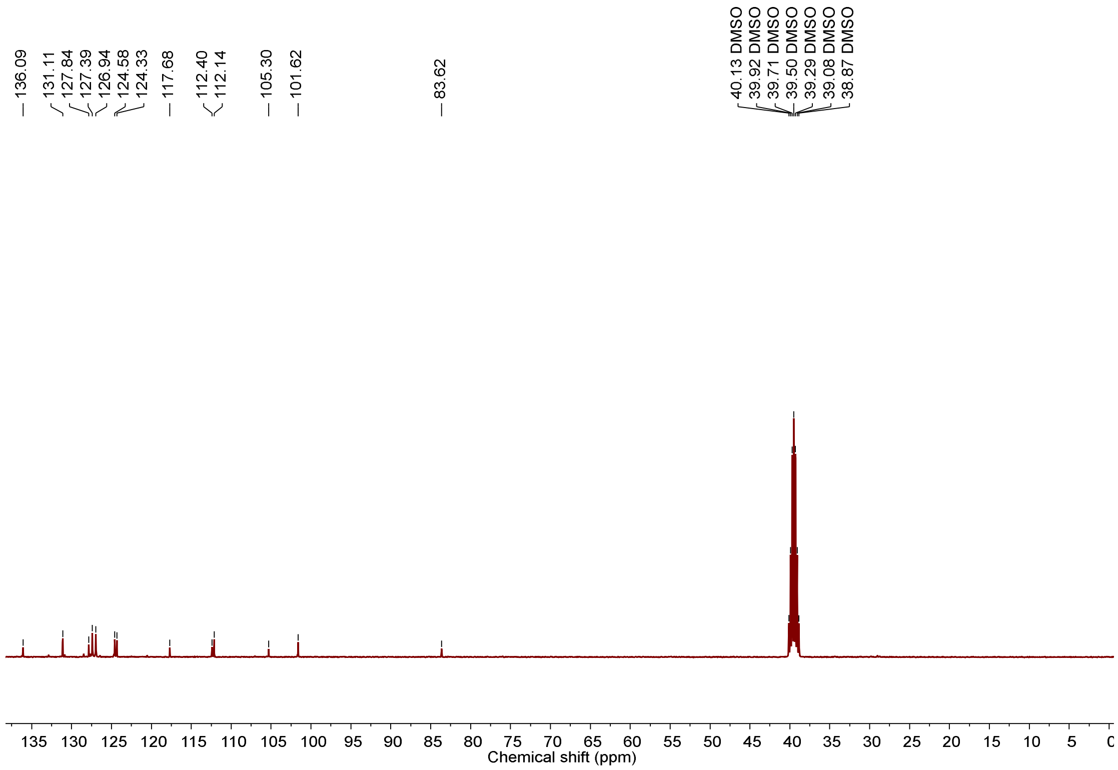

Supplementary Figure 61. ^{13}C NMR spectrum (100 MHz, DMSO-d_6, 298 K) of monomer **1**.

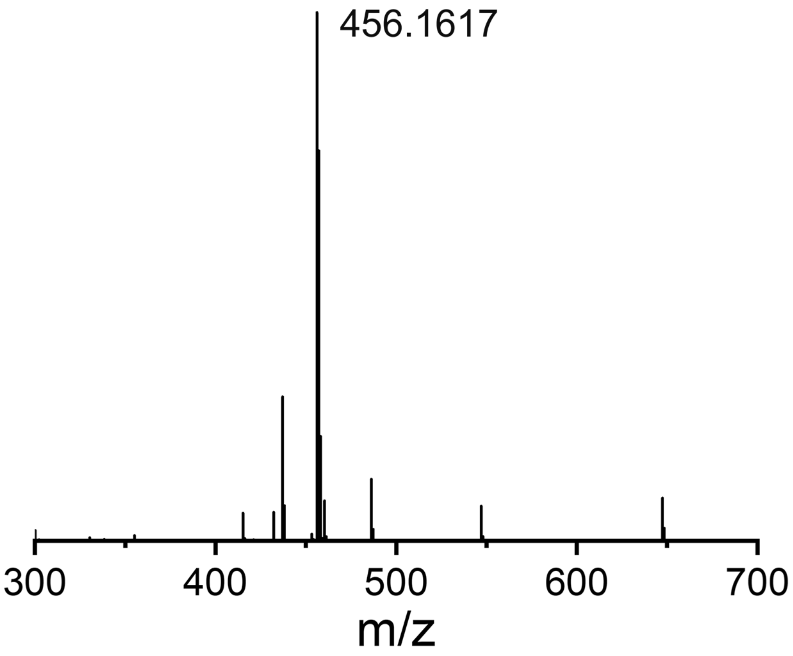

Supplementary Figure 62. HR-ESI-MS spectrum of monomer **1**.

Synthesis of monomer 2. To the mixture of 5-((10-ethynylanthracen-9-yl)ethynyl)-1H-indole (150 mg, 0.44 mmol, 1.0 equiv), iodobenzene (179.5 mg, 0.88 mmol, 2.0 equiv), CuI (1.9 mg, 0.01 mmol, 0.02 equiv), and $Pd(PPh_3)_4$ (11.6 mg, 0.01mmol, 0.01 equiv) were added THF (20 mL) and TEA (20 mL) under nitrogen atmosphere. The reaction mixture was stirred at 70 °C overnight under a nitrogen atmosphere. Upon completion, the solution was evaporated in vacuo to dryness. The crude product was purified by silica-gel column chromatography using mixed solvents of petroleum ether/dichloromethane (3:2, *v*/*v*) as the eluent to give a desired product in orange solid (150.1 mg, yield 82%). ^{1}H NMR (400 MHz, $CDCl_3$) δ (ppm) = 8.78 (dd, J = 6.4, 3.4 Hz, 2H), 8.70 (dd, J = 6.8, 3.0 Hz, 2H), 8.31 (s, 1H), 8.13 (s, 1H), 7.79 (d, J = 6.7 Hz, 2H), 7.65 (dt, J = 7.6, 3.8 Hz, 5H), 7.51 – 7.42 (m, 4H), 7.32 – 7.29 (m, 1H), 6.65 (s, 1H). ^{13}C NMR (101 MHz, DMSO-d_6) δ (ppm) = 136.14, 131.63, 131.41, 130.98, 129.29, 128.94, 127.82, 127.75, 127.46, 126.99, 126.73, 124.60, 124.43, 122.30, 118.83, 116.38, 112.23, 112.14, 105.80, 102.39, 101.62, 85.94, 83.51. ESI-MS (m/z): $[M]^+$ calcd 417.1512, found 417.1511.

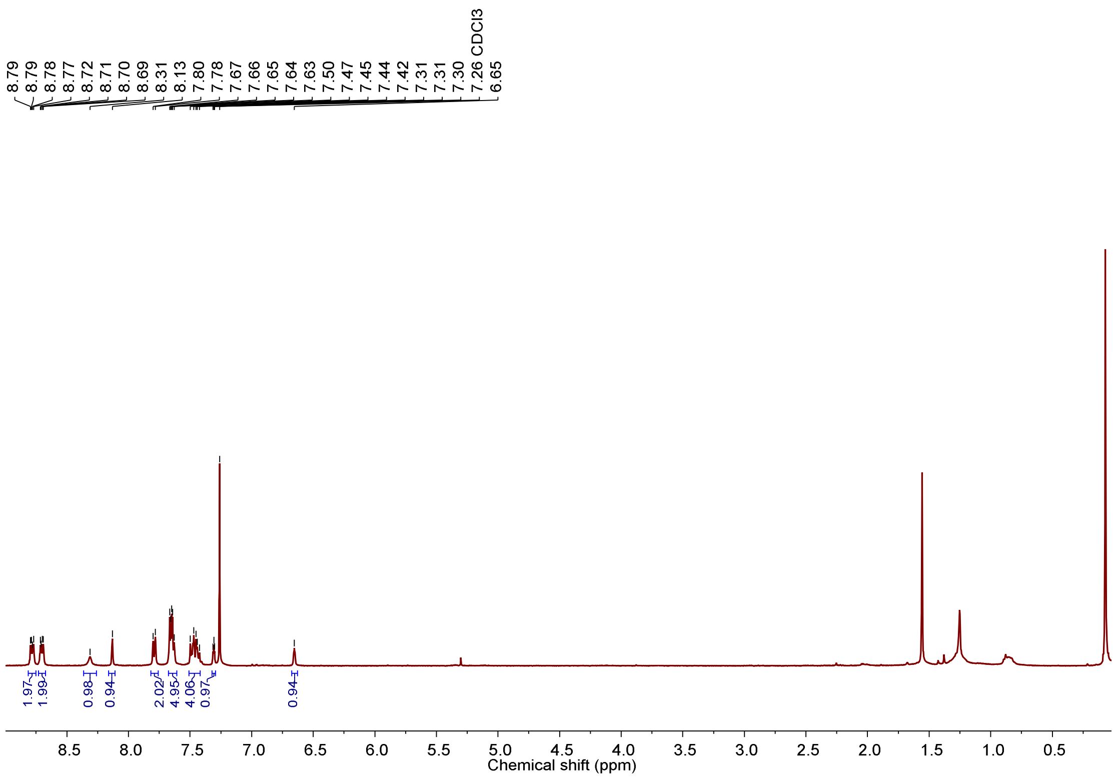

Supplementary Figure 63. ^{1}H NMR spectrum (400 MHz, $CDCl_3$, 298 K) of monomer **2**.

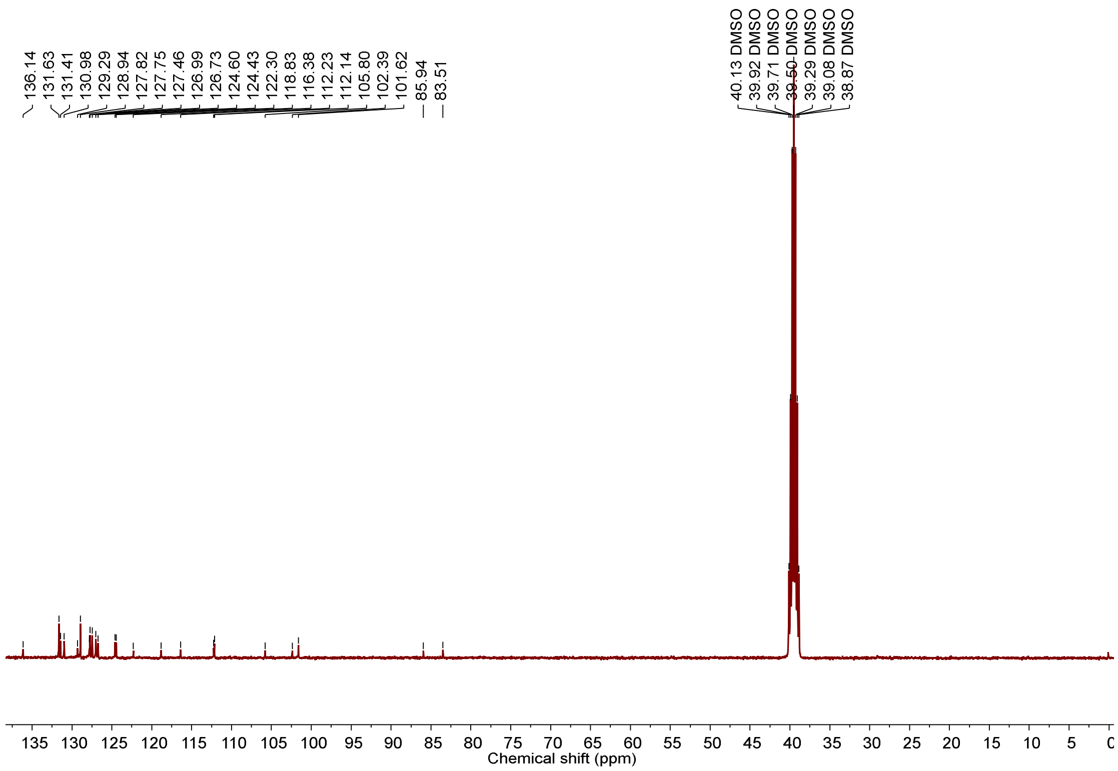

Supplementary Figure 64. ^{13}C NMR spectrum (100 MHz, DMSO-d_6, 298 K) of monomer **2**.

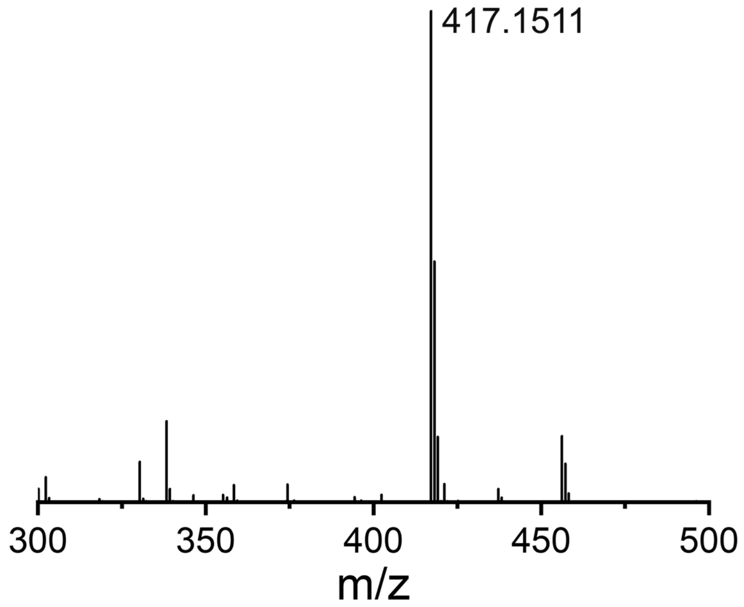

Supplementary Figure 65. HR-ESI-MS spectrum of monomer **2**.

3.3 Synthesis of monomer **3**

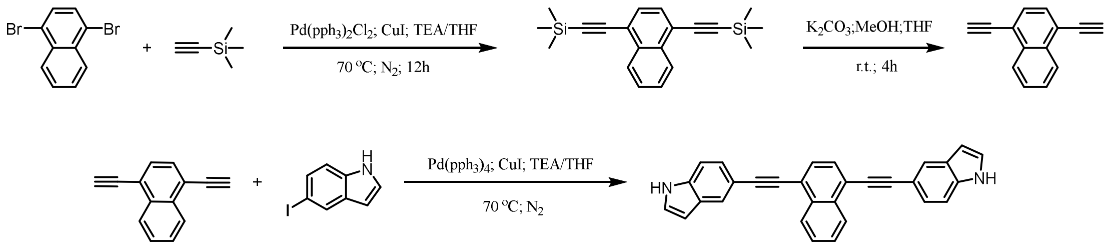

3

Supplementary Figure 66. The synthesis routes toward monomer **3**.

1,4-Diethynylnaphthalene. The mixture of 1,4-dibromonaphthalene (1.0 g, 3.5 mmol, 1 equiv), $Pd(PPh_3)_2Cl_2$ (49.2 mg, 0.07 mmol, 0.02 equiv) and CuI (26.7 mg, 0.14 mmol, 0.04 equiv) was added into THF (20 mL) and TEA (20 mL) under nitrogen atmosphere. Trimethylsilylacetylene (857 mg, 1.5mL, 8.8 mmol, 2.5 equiv) was added dropwise to the reaction mixture over 30 minutes. After stirring at 70 °C for 12 hours, the crude product was purified by silica-gel column chromatography using mixed solvents of petroleum ether/ethyl acetate (20:1, *v*/*v*) as the eluent to give a desired product in white solid. It was then treated with K_2CO_3 (856.91 mg, 6.2 mmol), KOH (347.82 mg, 6.2 mmol) in THF (20 mL) and MeOH (20 mL) at 25 °C for 4 hours. After the deprotection reaction, the crude product was purified by silica-gel column chromatography using mixed solvents of petroleum ether/dichloromethane (4:1, *v*/*v*) as the eluent to give a desired product in orange oil (542.1 mg, yield 87%). ^1H NMR (400 MHz, $CDCl_3$): δ (ppm) = 3.40 (s, 2H), 7.48 (dd, 2H), 7.53 (s, 2H). 8.35 (dd, 2H).

Synthesis of monomer 3. To the mixture of 1,4-diethynylnaphthalene (150.4 mg, 0.85 mmol, 1.0 equiv), 5-I-1H-indole (388.8 mg, 1.60 mmol, 2.0 equiv), CuI (1.9 mg, 0.01 mmol, 0.02 equiv), and $Pd(PPh_3)_4$ (11.6 mg, 0.01mmol, 0.01 equiv) were added THF (20 mL) and TEA (20 mL) under nitrogen atmosphere. The reaction mixture was stirred at 70 °C overnight under a nitrogen atmosphere. Upon completion, the solution was evaporated in vacuo to dryness. The crude product was purified by silica-gel column chromatography using mixed solvents of petroleum ether/dichloromethane (3:1, *v*/*v*) as the eluent to give a desired product in orange red solid (367.7 mg, yield 77%). ^1H NMR (400 MHz, DMSO-d_6) δ (ppm) = 11.41 (s, 2H), 8.50 (dd, J = 6.4, 3.3 Hz, 2H), 7.97 (s, 2H), 7.79 (d, J = 8.5 Hz, 4H), 7.50 (d, J = 8.4 Hz, 2H), 7.48–7.45 (m, 2H), 7.42 (d, J = 7.1 Hz, 2H), 6.53 (s, 2H). ^{13}C NMR (100 MHz, DMSO-d_6) δ (ppm) = 135.92, 132.33, 129.29,

127.74, 127.71, 126.83, 126.30, 124.45, 124.15, 120.83, 112.19, 112.01, 101.50, 98.57, 84.67. ESI-MS (m/z): [M]$^+$ calcd 406.1465, found 406.1463.

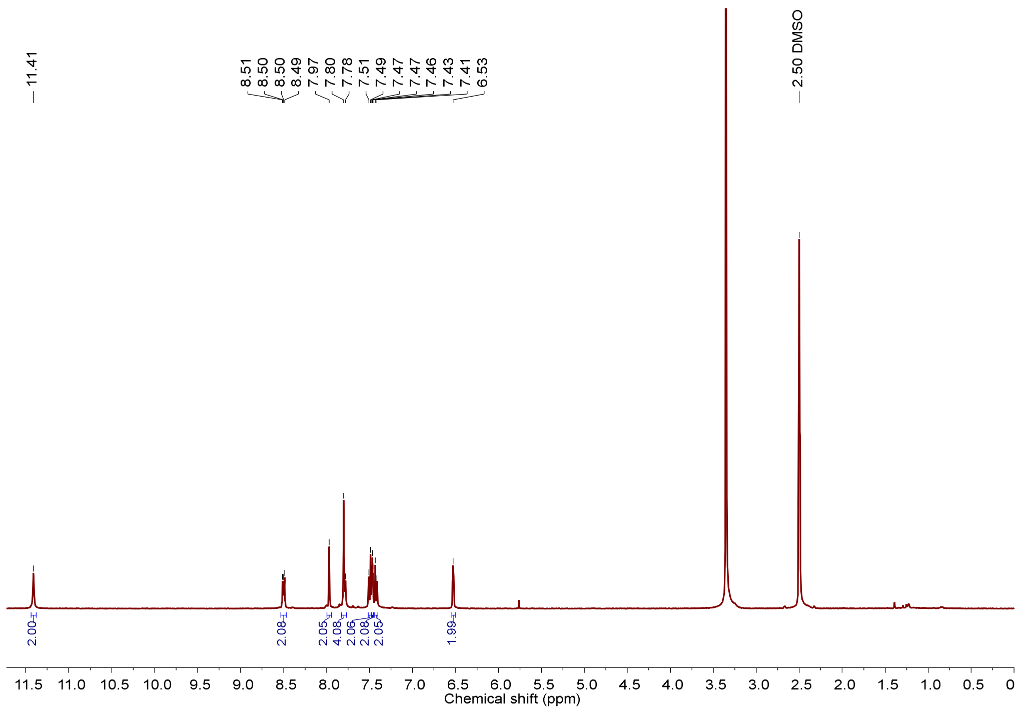

Supplementary Figure 67. ^{1}H NMR spectrum (400 MHz, DMSO-d_6, 298 K) of monomer **3**.

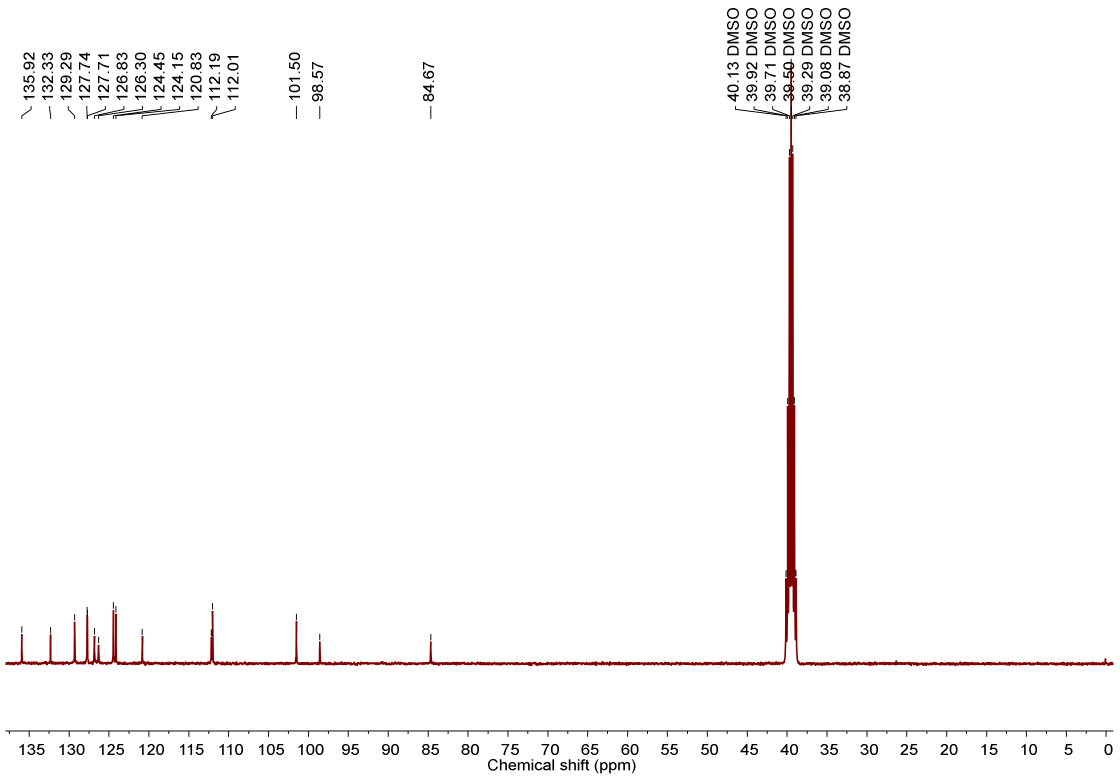

Supplementary Figure 68. ^{13}C NMR spectrum (100 MHz, DMSO-d_6, 298 K) of monomer **3**.

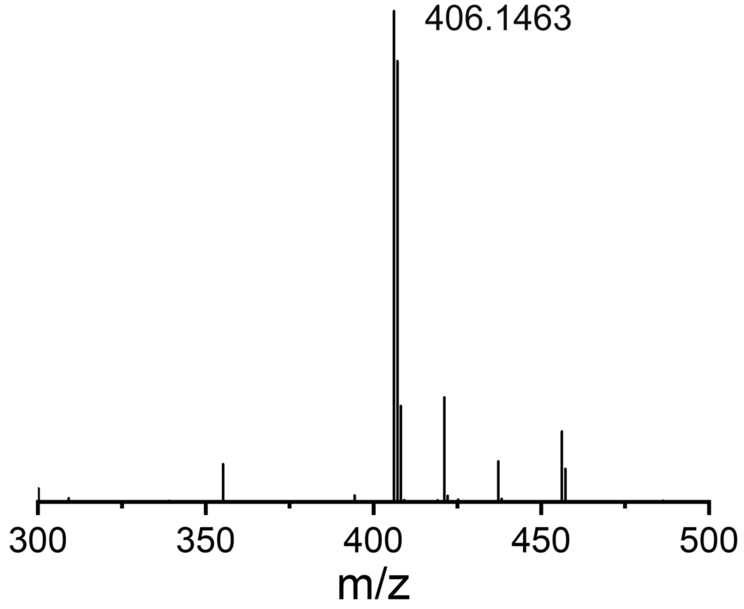

Supplementary Figure 69. HR-ESI-MS spectrum of monomer **3**.

3.4 Synthesis of monomer **4**

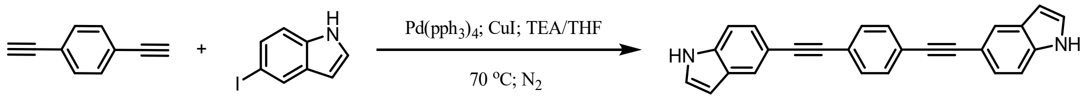

4

Supplementary Figure 70. The synthesis routes toward monomer **4**.

Synthesis of monomer 4. To the mixture of 1,4-diethynylbenzen (107.1 mg, 0.85 mmol, 1.0 equiv), 5-I-1H-indole (388.8 mg, 1.60 mmol, 2.0 equiv), CuI (1.9 mg, 0.01 mmol, 0.02 equiv), and $Pd(PPh_3)_4$ (11.6 mg, 0.01mmol, 0.01 equiv) were added THF (20 mL) and TEA (20 mL) under nitrogen atmosphere. The reaction mixture was stirred at 70 °C overnight under nitrogen atmosphere. Upon completion, the solution was evaporated in vacuo to dryness. The crude product was purified by silica-gel column chromatography using mixed solvents of petroleum ether/dichloromethane (3:1, *v*/*v*) as the eluent to give a desired product in yellow solid (41.3 mg, yield 14%). ^1H NMR (400 MHz, DMSO-d_6) δ (ppm) = 11.37 (s, 2H), 7.81 (s, 2H), 7.56 (s, 4H), 7.44 (dd, J = 5.9, 2.7 Hz, 4H),

7.27 (d, J = 8.4 Hz, 2H), 6.48 (s, 2H). ^{13}C NMR (100 MHz, DMSO-d_6) δ (ppm) = 135.79, 131.31, 127.62, 126.76, 124.34, 123.95, 122.65, 112.11, 111.93, 101.44, 93.57, 86.45. ESI-MS (m/z): $[M]^+$ calcd 356.1308, found 356.1308.

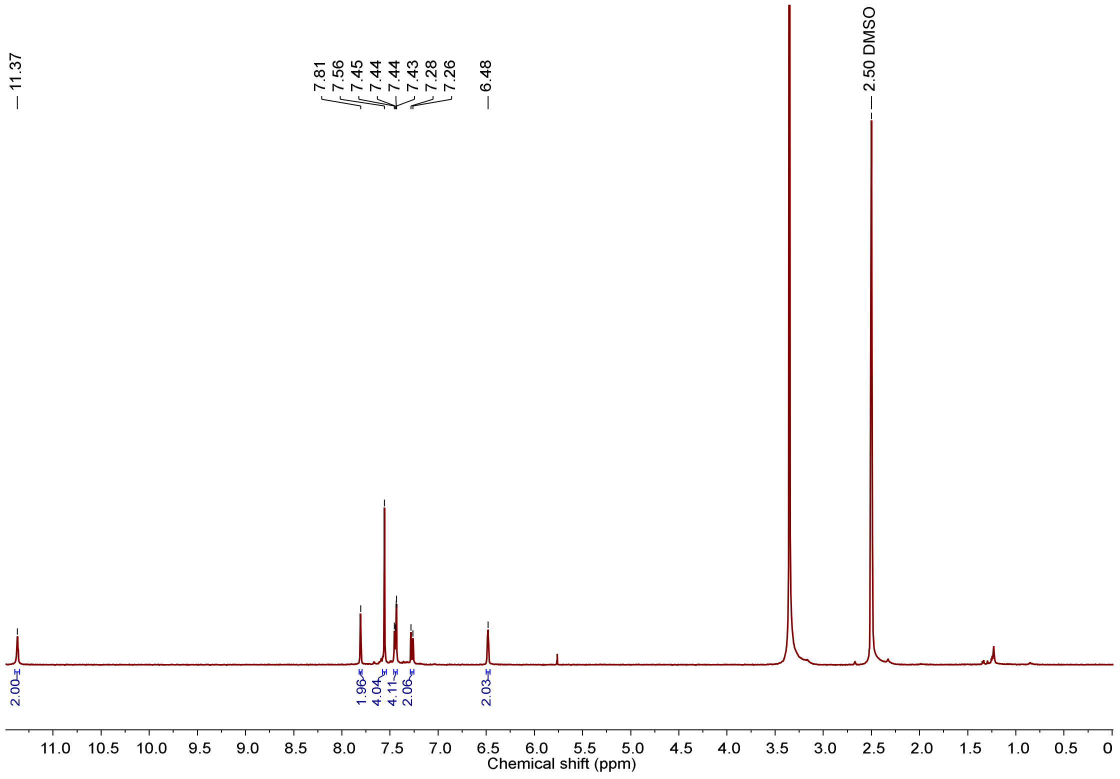

Supplementary Figure 71. ^{1}H NMR spectrum (400 MHz, DMSO-d_6, 298 K) of monomer **4**.

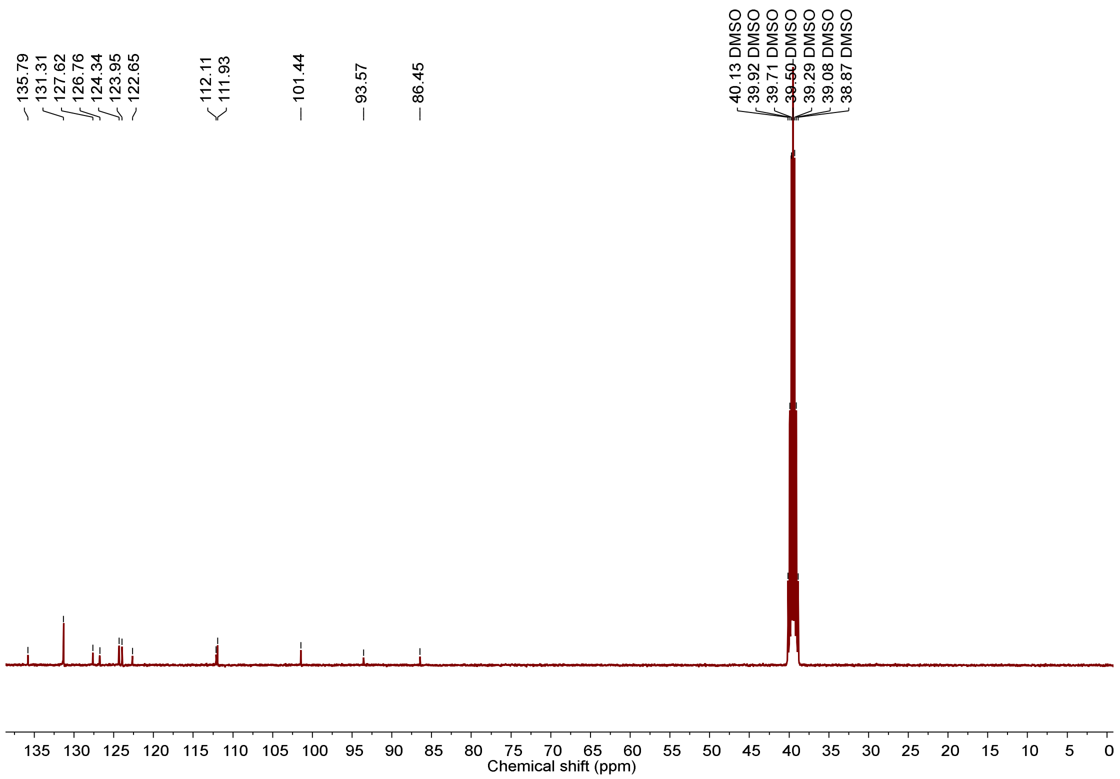

Supplementary Figure 72. ^{13}C NMR spectrum (100 MHz, DMSO-d_6, 298 K) of monomer **4**.

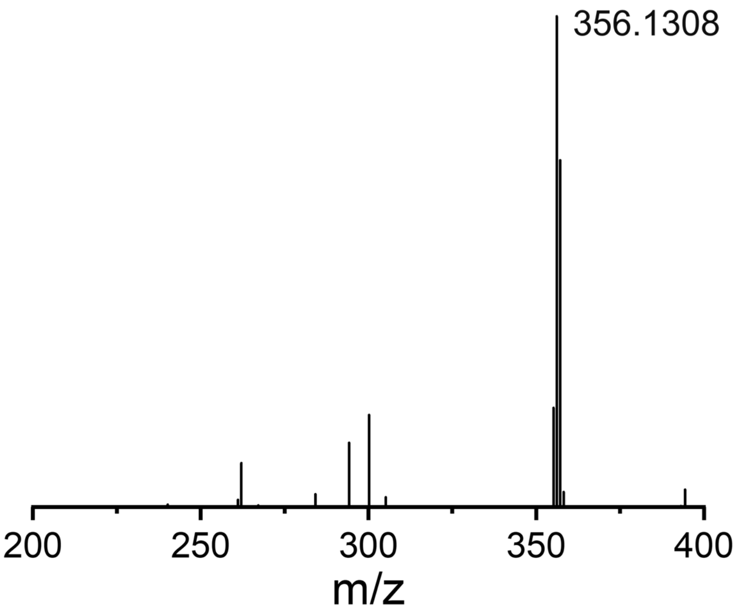

Supplementary Figure 73. HR-ESI-MS spectrum of monomer **4**.

3.5 Synthesis of monomer **5**

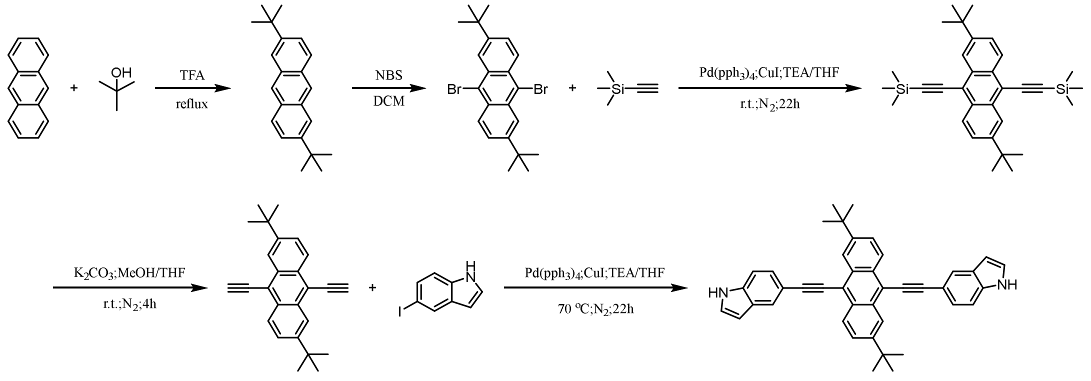

5

Supplementary Figure 74. The synthesis routes toward monomer **5**.

2,6-Di-tert-butylanthracene. A mixture of anthracene (1.78g, 10mmol), tert-butyl alcohol (2.22g, 30mmol), and TFA (10mL) was heated and reflow for 24 hours. The resulting dark brown solution was cooled to room temperature and water was added, then neutralized with sodium bicarbonate and extracted with petroleum ether. The ether layer was separated and dried with sodium sulfate and distilled under reduced pressure to give a brown solid (3.18g). It was then recrystalized with methanol to give a light yellow solid (2.19g, yield 75%). ^{1}H NMR (400 MHz, $CDCl_3$) δ (ppm) = 8.32 (s, 2H), 7.93 (d, J = 9.0 Hz, 2H), 7.86 (d, J = 1.9 Hz, 2H), 7.55 (dd, J = 9.0, 1.9 Hz, 2H), 1.44 (s, 18H).

9,10-Dibromo-2,6-di-tert-butylanthracene. 2,6-di-tert-butylanthracene (1.01 g, 3.48 mmol) and N-bromosuccinimide (1.24 g, 6.96 mmol) were added to a 150 mL Schlenk flask under a nitrogen atmosphere. Chloroform (30 mL) was then introduced, and the reaction mixture was stirred at room temperature for 24 hours. The reaction progress was monitored in real-time using thin-layer chromatography (TLC). After the reaction was complete, chloroform was removed under reduced pressure using a rotary evaporator. The reaction mixture was extracted three to four times with water and dichloromethane. The organic layer was collected, dried over anhydrous sodium sulfate, and the solvent was evaporated to yield the crude product. The crude product was further purified by silica gel column chromatography using petroleum ether/ethyl acetate (10:1) as the eluent, affording the desired white solid, 9,10-dibromo-2,6-di-tert-butylanthracene (1.47 g, yield 95%). ^{1}H NMR (400 MHz, $CDCl_3$) δ (ppm) = 8.51 (d, J = 9.3 Hz, 2H), 8.45 (d, J = 1.8 Hz, 2H), 7.71 (dd, J = 9.3, 1.9 Hz, 2H), 1.49 (s, 18H).

2,6-Di-tert-butyl-9,10-diethynylanthracene. Under a nitrogen atmosphere, a mixture of 9,10-dibromo-2,6-di-tert-butylanthracene (1.56 g, 3.5 mmol, 1 equiv), $Pd(PPh_3)_2Cl_2$ (49.2 mg, 0.07 mmol, 0.02 equiv), and CuI (26.7 mg, 0.14 mmol, 0.04 equiv) was added to THF (20 mL) and TEA (20 mL). After 30 minutes, trimethylsilylacetylene (857 mg, 1.5 mL, 8.8 mmol, 2.5 equiv) was slowly added dropwise to the reaction mixture. The reaction was stirred at room temperature for 22 hours, and the crude product was purified by silica gel column chromatography using a petroleum ether/ethyl acetate mixture (20:1, v/v) as the eluent to afford the desired product as a white solid. Subsequently, the product was treated with K_2CO_3 (856.91 mg, 6.2 mmol), KOH (347.82 mg, 6.2 mmol), THF (20 mL), and MeOH (20 mL) at 25°C for 4 hours. After the deprotection reaction, the crude product was purified by silica gel column chromatography using a petroleum ether/dichloromethane mixture (4:1, v/v) as the eluent, yielding the desired brown oily product (981.9 mg, 83% yield). Because the product was easy to deteriorate, no further characterization was carried out.

Synthesis of monomer 5. To the mixture of 2,6-di-tert-butyl-9,10-diethynylanthracene (170.3 mg, 0.5 mmol, 1.0 equiv), 5-I-1H-indole (243.1 mg, 1.0 mmol, 2.0 equiv), CuI (1.9 mg, 0.01 mmol, 0.02 equiv), and $Pd(PPh_3)_4$ (11.6 mg, 0.01mmol, 0.01 equiv) were added THF (20 mL) and TEA (20 mL) under nitrogen atmosphere. The reaction mixture was stirred at 70 °C overnight under nitrogen atmosphere. Upon completion, the solution was evaporated in vacuo to dryness. The crude product was purified by silica-gel column chromatography using mixed solvents of petroleum ether/dichloromethane (1:1, *v*/*v*) as the eluent to give a desired product in reddish brown solid (72.3 mg, yield 25%). ^{1}H NMR (400 MHz, $CDCl_3$) δ (ppm) =8.70 (dd, J = 5.3, 3.6 Hz, 4H), 8.29 (s, 2H), 8.09 (s, 2H), 7.74 (d, J = 10.8 Hz, 2H), 7.62 (d, J = 8.4 Hz, 2H), 7.48 (d, J = 8.4 Hz, 2H), 7.32–7.28 (m, 2H), 6.65 (s, 2H), 1.54 (s, 18H). ^{13}C NMR (100 MHz, DMSO-d_6) δ (ppm) = 149.06, 136.03, 130.78, 129.78, 127.87, 127.00, 126.64, 124.33, 123.96, 120.81, 116.87, 112.57, 112.29, 104.95, 101.61, 83.79, 40.13, 30.59. ESI-MS (m/z): $[M]^+$ calcd 568.2873, found 568.2869.

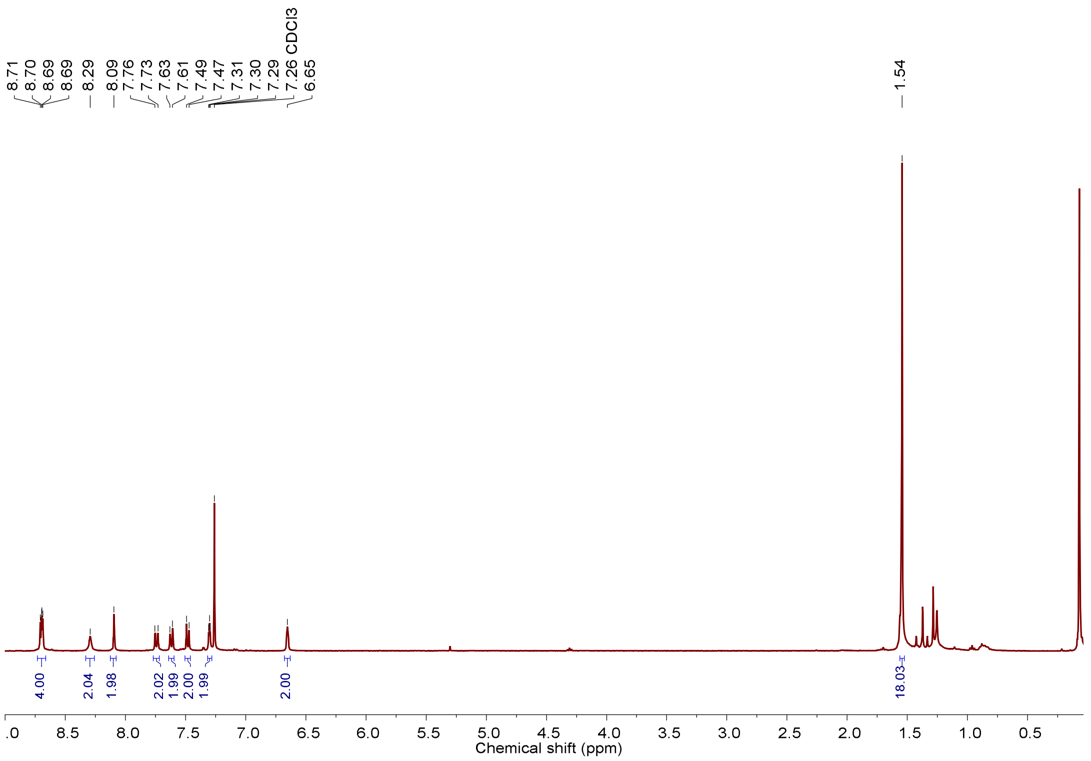

Supplementary Figure 75. ^{1}H NMR spectrum (400 MHz, $CDCl_3$, 298 K) of monomer **5**.

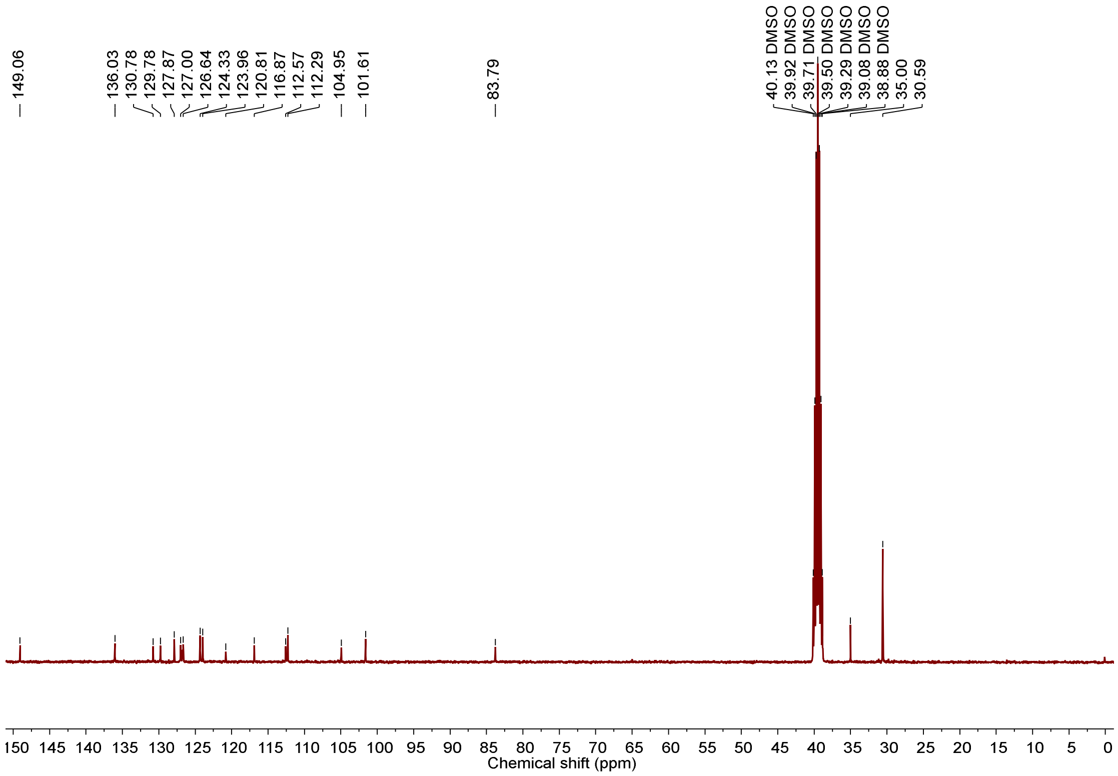

Supplementary Figure 76. ^{13}C NMR spectrum (100 MHz, DMSO-d_6, 298 K) of monomer **5**.

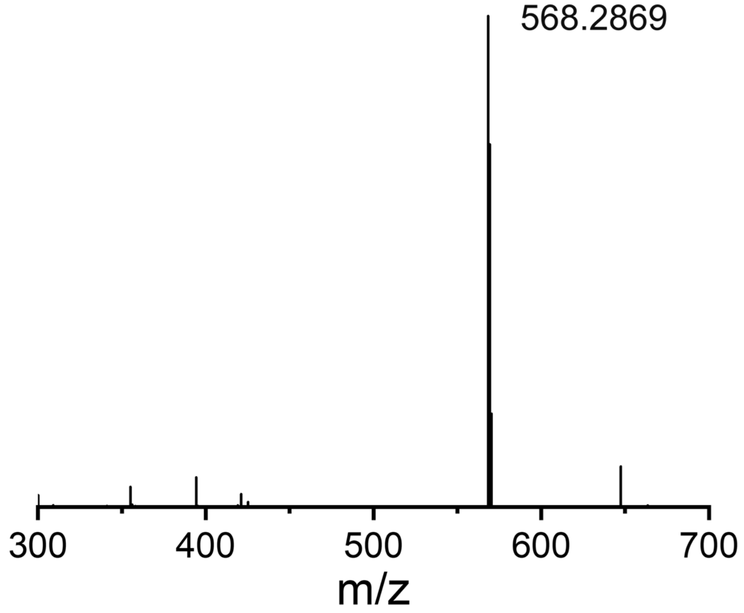

Supplementary Figure 77. HR-ESI-MS spectrum of monomer **5**.

4. Supplementary References

(1) Adelizzi, B. *et al*. Supramolecular Block Copolymers under Thermodynamic Control. *J. Am. Chem. Soc.* **140**, 7168 (2018).

(2) Bentea, L., Watzky, M. A. & Finke, R. G. Sigmoidal Nucleation and Growth Curves Across Nature Fit by the Finke−Watzky Model of Slow Continuous Nucleation and Autocatalytic Growth: Explicit Formulas for the Lag and Growth Times Plus Other Key Insights. *J. Phys. Chem. C* **121**, 5302 (2017).

(3) Smulders, M. M. J. *et al*. How to Distinguish Isodesmic from Cooperative Supramolecular Polymerisation. *Chem. Eur. J.* **16**, 362 (2010).
