# Supplementary material for: A coopetition-driven strategy of parallel/perpendicular aromatic stacking enabling metastable supramolecular polymerization
Source: Nat Commun. 2024 Dec 30;15:10762. doi: 10.1038/s41467-024-55106-z (PMC11686131; doi:10.1038/s41467-024-55106-z)
Supplement: Supplementary file 2 — Reporting Summary [file 41467_2024_55106_MOESM2_ESM.pdf]

nature portfolio

Corresponding author(s): Wei Tian

Last updated by author(s): Nov 26, 2024

Reporting Summary

Nature Portfolio wishes to improve the reproducibility of the work that we publish. This form provides structure for consistency and transparency in reporting. For further information on Nature Portfolio policies, see our Editorial Policies and the Editorial Policy Checklist.

Statistics

For all statistical analyses, confirm that the following items are present in the figure legend, table legend, main text, or Methods section.

n/a	Confirmed	
☐	☒	The exact sample size (*n*) for each experimental group/condition, given as a discrete number and unit of measurement
☐	☒	A statement on whether measurements were taken from distinct samples or whether the same sample was measured repeatedly
☐	☒	The statistical test(s) used AND whether they are one- or two-sided<br>*Only common tests should be described solely by name; describe more complex techniques in the Methods section.*
☒	☐	A description of all covariates tested
☒	☐	A description of any assumptions or corrections, such as tests of normality and adjustment for multiple comparisons
☐	☒	A full description of the statistical parameters including central tendency (e.g. means) or other basic estimates (e.g. regression coefficient) AND variation (e.g. standard deviation) or associated estimates of uncertainty (e.g. confidence intervals)
☐	☒	For null hypothesis testing, the test statistic (e.g. *F*, *t*, *r*) with confidence intervals, effect sizes, degrees of freedom and *P* value noted<br>*Give P values as exact values whenever suitable.*
☒	☐	For Bayesian analysis, information on the choice of priors and Markov chain Monte Carlo settings
☒	☐	For hierarchical and complex designs, identification of the appropriate level for tests and full reporting of outcomes
☒	☐	Estimates of effect sizes (e.g. Cohen's *d*, Pearson's *r*), indicating how they were calculated

Our web collection on statistics for biologists contains articles on many of the points above.

Software and code

Policy information about availability of computer code

Data collection	No software was used.
Data analysis	Origin 2023b 64 bit

For manuscripts utilizing custom algorithms or software that are central to the research but not yet described in published literature, software must be made available to editors and reviewers. We strongly encourage code deposition in a community repository (e.g. GitHub). See the Nature Portfolio guidelines for submitting code & software for further information.

Data

Policy information about availability of data

All manuscripts must include a data availability statement. This statement should provide the following information, where applicable:

- Accession codes, unique identifiers, or web links for publicly available datasets
- A description of any restrictions on data availability
- For clinical datasets or third party data, please ensure that the statement adheres to our policy

The data that support the findings of this study are available in the paper, Supplementary Information, Source data, and also from the authors on request. Crystallographic data have been deposited at the Cambridge Crystal-lographic Data Centre, under deposition numbers CCDC 2351020 (1AggII), 2351018 (2AggII),

2368744 (3AggII) and 2368743 (4). These data can be obtained free of charge from http://www.ccdc.cam.ac.uk/structures/. Source data are provided with this paper.

Research involving human participants, their data, or biological material

Policy information about studies with human participants or human data. See also policy information about sex, gender (identity/presentation), and sexual orientation and race, ethnicity and racism.

Reporting on sex and gender	This work was not involved in any human participants or human data. Only the A549 cell imaging experiments were conducted in vitro.
Reporting on race, ethnicity, or other socially relevant groupings	This work was not involved in any human participants or human data.
Population characteristics	This work was not involved in any human participants or human data.
Recruitment	This work was not involved in any human participants or human data.
Ethics oversight	This work was not involved in any human participants or human data.

Note that full information on the approval of the study protocol must also be provided in the manuscript.

Field-specific reporting

Please select the one below that is the best fit for your research. If you are not sure, read the appropriate sections before making your selection.

☒ Life sciences ☐ Behavioural & social sciences ☐ Ecological, evolutionary & environmental sciences

For a reference copy of the document with all sections, see nature.com/documents/nr-reporting-summary-flat.pdf

Life sciences study design

All studies must disclose on these points even when the disclosure is negative.

Sample size	No statistical methods were used to predetermine sample size. For clearly see the cell images in the field of vision in the confocal laser scanning microscopy, A549 and B16-F10 cells were seeded at a density of 5 × 103 cells per well in 96-well plates.
Data exclusions	No data were excluded from the analyses.
Replication	Cell imaging experiments were repeated for three times, and we confirmed that the attempts at replication were successfully achieved.
Randomization	The cell samples were random allocated into the experimental groups.
Blinding	The investigators were blinded to group allocation during data collection and analysis.

Reporting for specific materials, systems and methods

We require information from authors about some types of materials, experimental systems and methods used in many studies. Here, indicate whether each material, system or method listed is relevant to your study. If you are not sure if a list item applies to your research, read the appropriate section before selecting a response.

Materials & experimental systems

n/a	Involved in the study	
☒	☐	Antibodies
☐	☒	Eukaryotic cell lines
☒	☐	Palaeontology and archaeology
☒	☐	Animals and other organisms
☒	☐	Clinical data
☒	☐	Dual use research of concern
☒	☐	Plants

Methods

n/a	Involved in the study	
☒	☐	ChIP-seq
☒	☐	Flow cytometry
☒	☐	MRI-based neuroimaging

Eukaryotic cell lines

Policy information about cell lines and Sex and Gender in Research

Cell line source(s)	The human lung adenocarcinoma epithelial cell line A549 (male, CCL-185) was obtained from the Chinese Academy of Science Cell Bank, and B16-F10 cell line (ATCC, CRL-6475) was purchased from Wuhan Pricella Biotechnology Co., Ltd..
Authentication	All cell lines were authenticated by short tandem repeat profiling. B16-F10 cells were also spot checked as they express melanin and cell pellets are black (no other cell lines in use are black when pelleted).
Mycoplasma contamination	All cell lines were tested negative for mycoplasma.
Commonly misidentified lines (See ICLAC register)	No commonly misidentified cell lines were used in the study.

Plants

Seed stocks	No seeds were used in this work, and seed stocks or other plant material were not used.
Novel plant genotypes	No plants were used in this work, and no plant genotypes were produced.
Authentication	This work is not involved in any seed stocks and novel plant genotypes.
